# Multimarker Proteomic Profiling for the Prediction of Cardiovascular Mortality in Patients with Chronic Heart Failure

**DOI:** 10.1371/journal.pone.0119265

**Published:** 2015-04-23

**Authors:** Gilles Lemesle, Fleur Maury, Olivia Beseme, Lionel Ovart, Philippe Amouyel, Nicolas Lamblin, Pascal de Groote, Christophe Bauters, Florence Pinet

**Affiliations:** 1 INSERM UMR1167, Lille, France; 2 Institut Pasteur de Lille, Lille, France; 3 Université Lille Nord de France, Lille, France; 4 Centre Hospitalier Régional et Universitaire de Lille, Lille, France; 5 Faculté de Médecine de Lille, Lille, France; I2MC INSERM UMR U1048, FRANCE

## Abstract

Risk stratification of patients with systolic chronic heart failure (HF) is critical to better identify those who may benefit from invasive therapeutic strategies such as cardiac transplantation. Proteomics has been used to provide prognostic information in various diseases. Our aim was to investigate the potential value of plasma proteomic profiling for risk stratification in HF. A proteomic profiling using surface enhanced laser desorption ionization - time of flight - mass spectrometry was performed in a case/control discovery population of 198 patients with systolic HF (left ventricular ejection fraction <45%): 99 patients who died from cardiovascular cause within 3 years and 99 patients alive at 3 years. Proteomic scores predicting cardiovascular death were developed using 3 regression methods: support vector machine, sparse partial least square discriminant analysis, and lasso logistic regression. Forty two ion m/z peaks were differentially intense between cases and controls in the discovery population and were used to develop proteomic scores. In the validation population, score levels were higher in patients who subsequently died within 3 years. Similar areas under the curves (0.66 – 0.68) were observed for the 3 methods. After adjustment on confounders, proteomic scores remained significantly associated with cardiovascular mortality. Use of the proteomic scores allowed a significant improvement in discrimination of HF patients as determined by integrated discrimination improvement and net reclassification improvement indexes. In conclusion, proteomic analysis of plasma proteins may help to improve risk prediction in HF patients.

## Introduction

In spite of recent therapeutic improvements, chronic heart failure (HF) remains a major public health problem [1,2] with a high rate of mortality [3]. Risk stratification is a critical issue in patients with systolic HF since high-risk patients can therefore be considered for invasive strategies such as implantable assist devices and/or cardiac transplantation. Variables such as New-York Heart Association (NYHA) class, left ventricular ejection fraction (LVEF), brain natriuretic peptide (BNP), or variables obtained during cardiopulmonary exercise testing (peak oxygen consumption (peak VO_2_)) have been associated with the outcome of HF patients [4,5,6,7]. In spite of these advances, risk stratification of HF patients needs to be further improved. Indeed, there remains variability in the prognosis with some patients who are categorized at low risk but experience early mortality; and conversely, patients categorized as severe but have an unexpectedly prolonged survival.

There is a need for novel prognostic markers that may help to better stratify the risk of major cardiac events in HF patients. Recently, a systematic review of 55 papers dealing with risk prediction model accuracy has only shown a moderate succesfulness for prediction of mortality in HF patients using “classic” prognostic evaluation and emphasized the need for models using a systematic biology approach [8]. Due to their availability and non-invasive nature, circulating biomarkers are currently the subject of intense research in this area [9,10]. Surface enhanced laser desorption ionization—time of flight—mass spectrometry (SELDI-TOF-MS), a proteomic technology which is a combination of chromatography on proteinchip arrays and mass spectrometry, offers a high throughput non a priori strategy for the detection of differentially expressed biomarkers [11]. This may thus allow developing a multimarker strategy for improving risk prediction in HF patients.

The aim of the present study was to investigate the potential value of plasma proteomic profiling for risk stratification in HF. Proteomic scores predictive of cardiovascular mortality were developed in a discovery population of chronic HF patients; then, their performances were tested in a validation cohort and challenged against established prognostic indicators.

## Methods

### Population

All patients referred for evaluation of systolic HF (LVEF <45%) in our institution between November 1998 and May 2010 have been included in a prospective cohort on prognostic indicators [6,12,13,14]. The study was approved by the ethics committee of the Centre Hospitalier de Lille (Lille, France) and complies with the Declaration of Helsinski. All patients gave written informed consent. All patients were ambulatory and clinically stable for at least 2 months, and received optimal medical therapy with maximal tolerated doses of angiotensin-converting enzyme inhibitors and betablockers. At inclusion, patients underwent a prognostic evaluation including: BNP level assessment, echocardiography, and cardiopulmonary exercise testing as previously described [6,13]. In addition, patients underwent a coronary angiogram to help define the etiology of left ventricular (LV) systolic dysfunction as either ischemic or non-ischemic. A follow-up was performed at 3 years to assess clinical outcome. All events were adjudicated by two investigators and by a third one in case of disagreement. Cardiovascular death included cardiovascular-related death, urgent transplantations defined as United Network for Organ Sharing (UNOS) status 1), and urgent assist device implantation.

**Fig 1:**
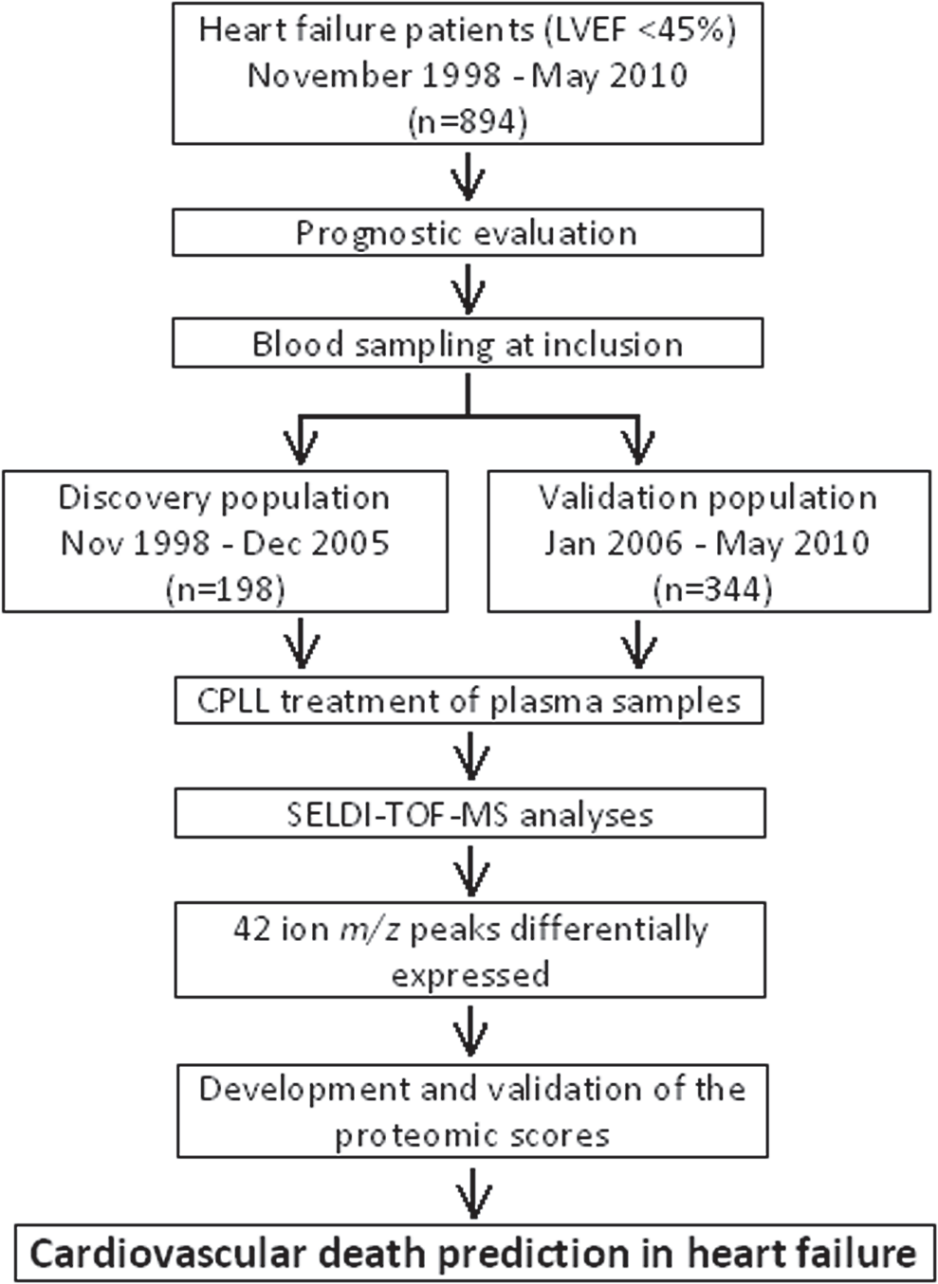
Flow chart of the study. Overview of the study design and analyses performed to build the proteomic scores and to test their validation. A Bonferroni correction was applied on the ion m/z peaks detected by SELDI-TOF analysis. Three different statistical regression methods (SVM, sPLS-DA and LASSO) were used to build the scores with the 42 differentially intense ion m/z peaks. The performance of the proteomic scores was then tested. LVEF: left ventricular ejection fraction, CPLL: Combinatorial peptide ligand library, SELDI-TOF-MS: Surface enhanced laser desorption ionization—time of flight—mass spectrometry.

The flow chart of the present study is shown in Fig 1. For the discovery phase, we selected 198 patients included between November 1998 and December 2005. Ninety nine patients who died from cardiovascular cause within 3 years after the initial prognostic evaluation (cases) were individually matched for age, sex, and HF etiology with 99 patients who were still alive at 3 years (controls). For the validation phase, the proteomic analysis was repeated in a population of 344 consecutive patients included between January 2006 and May 2010.

### Proteomic analyses of plasma samples

Detailed process is described in supplemental methods (see S1 Methods). At inclusion, peripheral blood was collected in tubes containing EDTA and plasma samples were stored at -80°C. Prior to the proteomic study, plasma samples underwent no more than two freeze/thaw cycles. One mL of each plasma sample was treated with the ProteoMiner protein enrichment kit (Bio-Rad Laboratories, Hercules, CA, USA) as previously described [15,16]. This combinatorial peptide ligand library (CPLL) method has been shown to be reproducible and allow access to proteolytic fragments [11].

Proteomic analyses were performed on CPLL-treated plasma samples in both populations using the SELDI-TOF-MS technique. CPLL-treated plasma samples were profiled with eight-spot format CM10 (Weak Cation Exchanger) and H50 (Reverse Phase) ProteinChip arrays (Bio-Rad Laboratories). To obtain ion m/z peak intensities, all arrays were read in an automated PBS 4000 SELDI-TOF-MS (Bio-Rad Laboratories) as previously described [11]. Samples were analyzed in duplicate and randomly distributed on arrays. Representative mass spectra are displayed in S1 Fig.

### Statistical analyses

All statistical analyses were performed using R Statistical Package version 3.0. Continuous variables are presented as mean ± standard deviation (SD) and were compared using Student’s t-test. Categorical variables are expressed as absolute number and/or percentages and were compared using the χ2 test or the Fisher test as appropriate. Single imputation was used for clinical and proteomic missing data. In the discovery set, missing proteomic data (1 patient) were imputed with the median of the corresponding ion m/z peak intensity. In the validation set, 9 peak VO_2_ values and 2 BNP values were imputed with their respective medians.

Proteomic variables were standardized before further analyses by subtracting the mean then dividing by the SD to have a mean of 0 and a SD of 1. Detailed analysis is described in the supplemental methods (see S2 Methods). The mean intensity of each ion m/z peak was compared between cases and controls with a Bonferroni correction to account for multiple testing. Three different statistical regression methods were applied on the selected ion m/z peaks in the discovery set to calculate proteomic scores predictive of cardiovascular mortality: the support vector machine (SVM), the sparse partial least square discriminant analysis (sPLS-DA), and a lasso logistic regression (LASSO). We used the following R packages: “kernlab” R package (version 0.9–19) for SVM [17], “spls” R package (version 2.2–1) for sPLS-DA [18] and “glmnet” R package (version 1.9–5) for LASSO [19]. The same 3 models were applied in the validation cohort to compute the predicted probabilities of cardiovascular death. Receiver operating characteristic (ROC) curve analysis was used to display the performance of the proteomic scores. Multivariate logistic regressions relating cardiovascular mortality to the proteomic scores were performed to calculate odds ratios (OR) and corresponding 95% confidence intervals (CI). Covariables included in final logistic regression models were: age, sex, HF etiology, diabetes, creatinine, NYHA class, BNP, LVEF and peak VO_2_. Finally, the incremental value of the proteomic scores to predict the cardiovascular mortality risk, when added to models with established prognostic indicators, was estimated with the continuous net reclassification improvement (NRI), and the integrated discrimination improvement (IDI).

## Results

**Fig 2:**
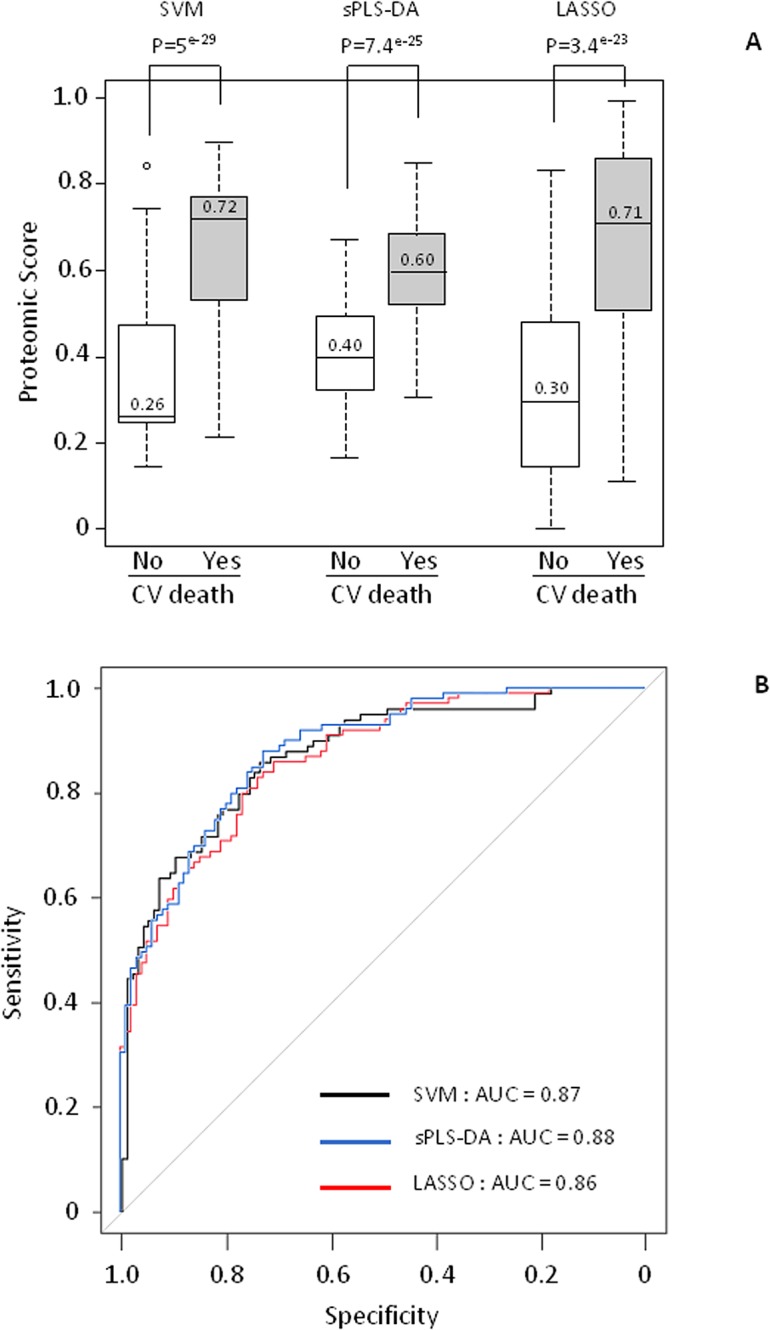
Proteomic score values and ROC curves in the discovery population. A: Three different regression methods (SVM, sPLS-DA and LASSO) were applied on the 42 ion m/z peaks differentially intense between cases and controls to calculate proteomic scores. The dark line inside the box plot indicates the median value whereas the extremities represent 75^th^ and 25^th^ percentiles. The whiskers above and below the dotted lines represent the maximum and minimum values except for outliers (either ≥ 1.5 times above the 3^rd^ quartile or ≤ 1.5 times below the 1^st^ quartile) that are represented by circles. B: ROC curves for performance of the proteomic scores. AUC indicates area under the curve.

Table 1 shows the baseline characteristics of the patients included in the discovery population. The patients who died from a cardiovascular cause during the 3-year follow-up showed significantly higher NYHA class, BNP level, creatinine level, and lower peak VO_2_ as compared to patients alive at 3 years. A total of 203 different ion m/z peaks was detected and analyzed by SELDI-TOF-MS in the CPLL-treated plasma of these patients (S1 Table). The 42 ion m/z peaks found to be differentially intense after Bonferroni correction (highlighted in blue in S1 Table) were used to build the proteomic scores. Sixteen of these peaks were highly correlated with correlation coefficients >0.9 (S2 Table) requiring feature selection in model construction. As shown in Fig 2A, the values of the proteomic scores obtained with the 3 statistical methods were consistently and significantly higher in cases as compared to controls. The ROC curves are shown in Fig 2B. High and similar areas under the curves (AUC) values (0.86–0.88) were observed. The 3 proteomic scores were highly correlated (S3 Table).

**Table 1:** Baseline characteristics of the patients included in the discovery population.

	DISCOVERY POPULATION		
	Cardiovascular death (n = 99)	No cardiovascular death(n = 99)	P value
Age (years)	58.6 ± 10.9	58.9 ± 10.6	na
Male	91	91	na
HF etiology			na
Ischemic	58	58	
Non ischemic	41	41	
Diabetes mellitus	34	33	0.901
NYHA class			0.004
1	1	8	
2	62	73	
3	36	18	
LV ejection fraction (%)	27.8 ± 9.9	28.7 ± 9.2	0.490
Peak VO_2_ (ml/min/kg)	13.5 ± 3.7	17.2 ± 4.9	<0.0001
BNP ^a^			0.002
Low	17	40	
Intermediate	43	33	
High	35	23	
Creatinine (mg/L)	12.5 ± 3.5	11 ± 2.6	0.0006
Treatment at inclusion			
ACE/ARB inhibitors	92	92	1
ß-blockers	90	94	0.407
Diuretics	87	77	0.06

na = non applicable

^a^ In the discovery population, BNP was measured by either a radio-immuno-assay (Shionoria BNP kit, Shionogi & Co. Ltd., Osaka, Japan) from 1998 to 2003 or by the Triage BNP assay (Biosite diagnostics Inc., San Diego, CA, USA) from 2003 to 2005. The BNP level was categorized as low (deciles 1, 2 and 3), intermediate (deciles 4,5, 6 and 7) or high (deciles 8, 9 and 10) for each individual patient.

**Fig 3:**
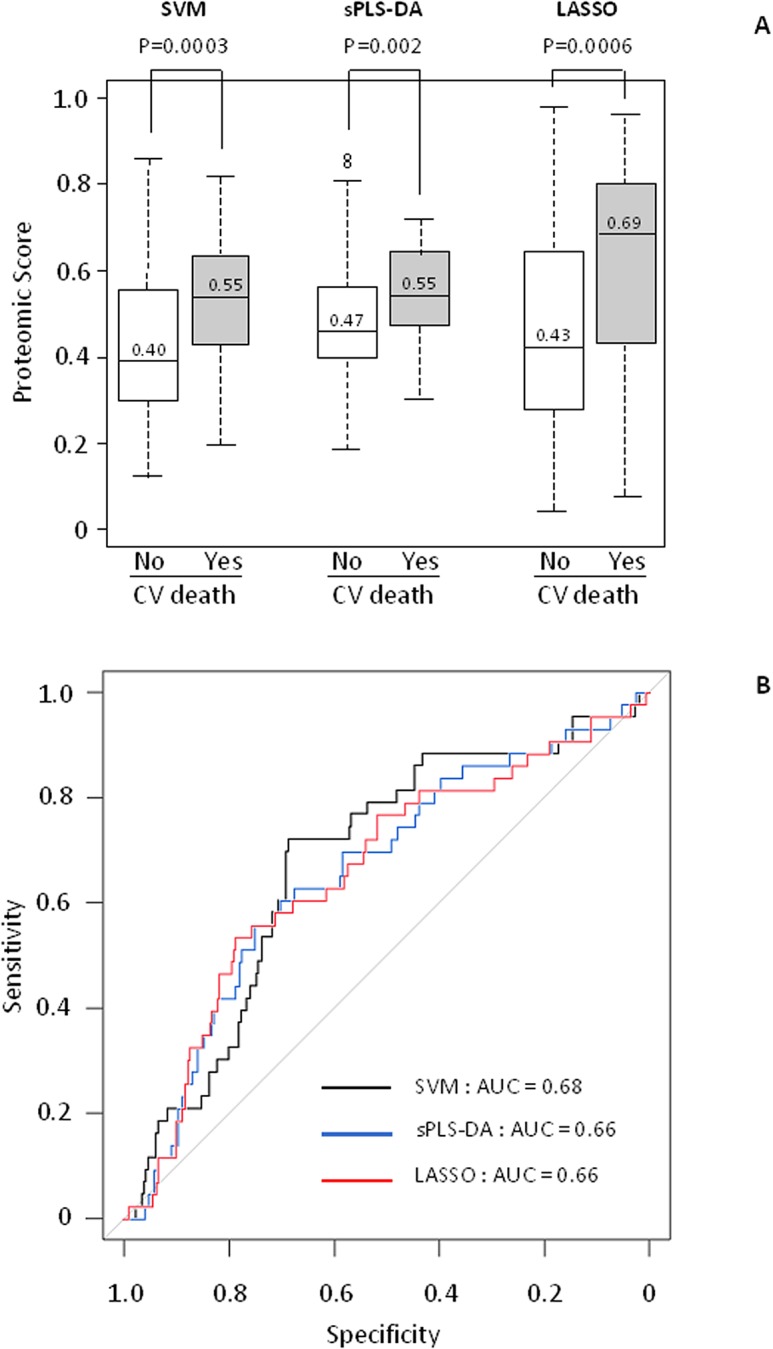
Proteomic score values and ROC curves in the validation population. A: The same regression methods (SVM, sPLS-DA and LASSO) were applied on the same 42 ion m/z peaks to calculate proteomic scores in the validation population. The dark line inside the box plot indicates the median value whereas the extremities represent 75^th^ and 25^th^ percentiles. The whiskers above and below the dotted lines represent the maximum and minimum values except for outliers (either ≥ 1.5 times above the 3^rd^ quartile or ≤ 1.5 times below the 1^st^ quartile) that are represented by circles. B: ROC curves for performance of the proteomic scores. AUC indicates area under the curve.

**Fig 4:**
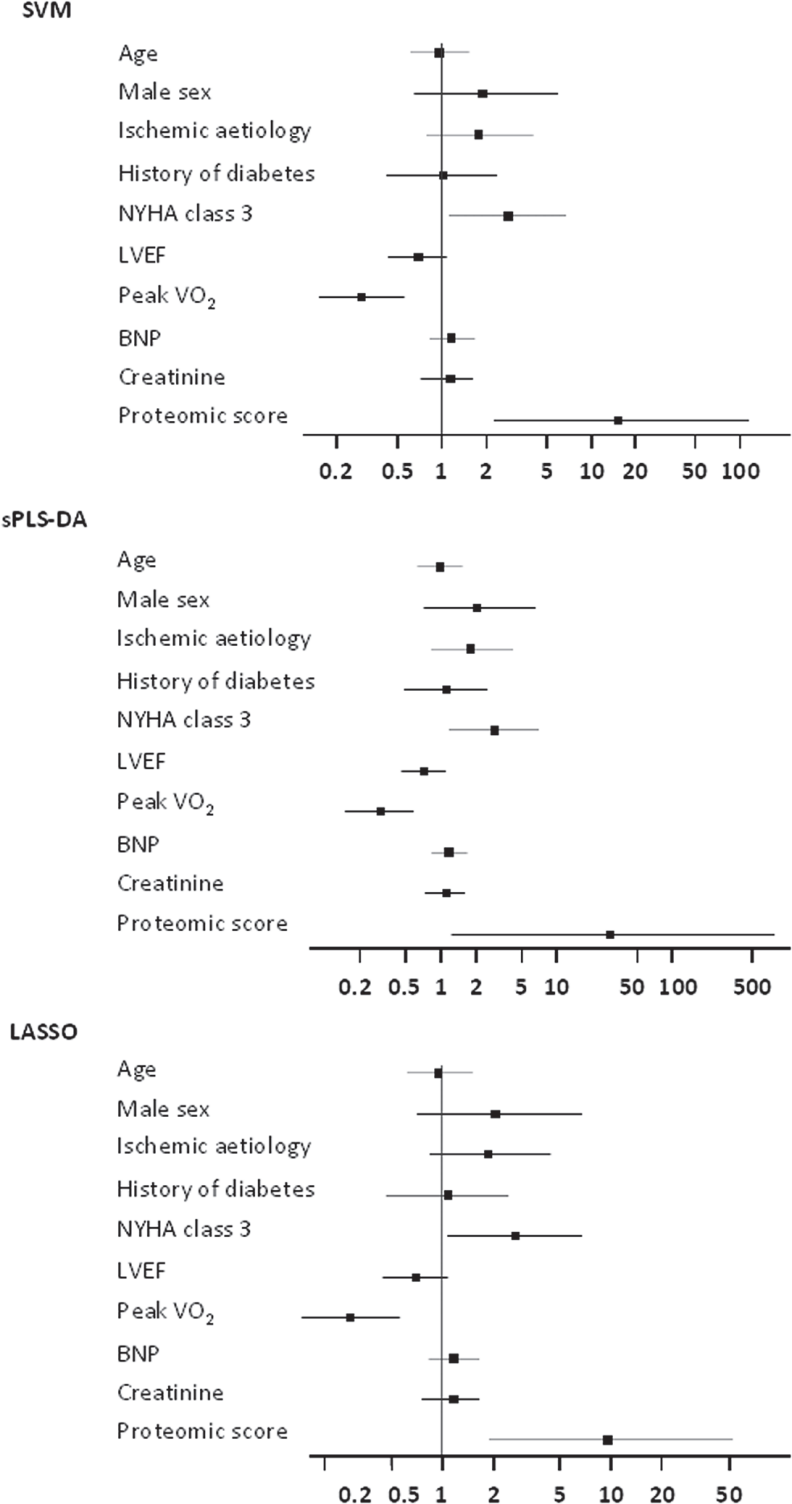
Independent predictors of cardiovascular death in the validation study. Data are odds ratios and 95% confidence intervals.

The proteomic scores were then tested in the validation population. Patients with non-cardiovascular death and patients with non-urgent heart transplantation (n = 35) were excluded from the analysis. The remaining 266 patients with no event were compared to the 43 patients with cardiovascular death during the 3-year follow-up period. As shown in Table 2, patients who died from a cardiovascular cause had more often ischemic HF, higher NYHA class and BNP, and lower LVEF and peak VO_2_. The values of the 3 proteomic scores were significantly higher in patients who died (Fig 3A). ROC curves are shown in Fig 3B; similar and modest AUC values (0.66–0.68) were found. As shown in Fig 4, after adjustment on confounders (age, sex, HF etiology, diabetes mellitus, creatinine, NYHA class, BNP, LVEF and peak VO_2_), the proteomic scores were still significantly associated with cardiovascular death in the validation population whatever the statistical method used (OR = 15.1 [2.2–112.9], P = 0.007 for SVM, OR = 29.4 [1.2–765.1], P = 0.03 for sPLS-DA and OR = 9.6 [1.9–59.1], P = 0.007 for LASSO). Other variables kept into the models were NYHA class and peak VO_2_. Finally, continuous NRI and IDI demonstrated that the proteomic scores calculated with the three methods significantly improved the prediction of cardiovascular death in HF patients (Table 3).

**Table 2:** Baseline characteristics of the patients included in the validation population.

	DISCOVERY POPULATION		
	Cardiovascular death (n = 99)	No cardiovascular death(n = 99)	P value
Age (years)	57 ± 12.6	54 ± 10.8	0.101
Male	33	214	0.885
HF etiology			0.011
Ischemic	28	118	
Non ischemic	15	148	
Diabetes mellitus	14	66	0.282
NYHA class			0.0005
1	0	32	
2	25	211	
3	18	23	
LV ejection fraction (%)	28.4 ± 9	35 ± 9.1	<0.0001
Peak VO_2_ (ml/min/kg)	13 ± 3.4	18.7 ± 5.9	<0.0001
BNP (pg/mL) ^a^	735 ± 757	238 ± 384	<0.0001
Creatinine (mg/L)	12.7 ± 4.9	11.1 ± 6.4	0.11
Treatment at inclusion			
ACE/ARB inhibitors	43	258	0.605
ß-blockers	40	254	0.448
Diuretics	40	195	0.005

^a^ In the validation population, BNP was measured using the Advia Centaur BNP assay (Bayer Healthcare LLC, Tarrytown, NY, USA) in all patient.

**Table 3:** Incremental value of the proteomic scores for the prediction of cardiovascular death.

Methods	SVM		SPLS-DA		LASSO	
	Mean ± SE (95% CI)	P value	Mean ± SE (95% CI)		Mean ± SE (95% CI)	P value
Net Reclassification Improvement (NRI)	0.66 ± 0.17 (0.33–0.99)	<0.0001	0.51 ± 0.17 (0.18–0.84)	Net Reclassification Improvement (NRI)	0.66 ± 0.17 (0.33–0.99)	<0.0001
NRI.event	0.33 ± 0.15 (0.03–0.64)	0.031	0.24 ± 0.15 (-0.06–0.54)	NRI.event	0.33 ± 0.15 (0.03–0.64)	0.031
NRI.non-event	0.33 ± 0.06 (0.21–0.45)	<0.0001	0.27 ± 0.06 (0.15–0.40)	NRI.non-event	0.33 ± 0.06 (0.21–0.45)	<0.0001
Integrated Discrimination Improvement (IDI)	0.03 ± 0.01 (0.01–0.06)	0.019	0.02 ± 0.01 (-0.01–0.04)	Integrated Discrimination Improvement (IDI)	0.03 ± 0.01 (0.01–0.06)	0.019

NRI is dichotomized as NRI.event (corresponding to the capacity of the proteomic score to reclassify those patients who died from cardiovascular cause) and NRI.non-event (corresponding to the capacity of the proteomic score to reclassify those alive patients).

## Discussion

It has been emphasized by recent international guidelines that the assessment of prognosis is an important step for the management of chronic systolic HF, particularly when counselling patients about devices and cardiac transplantation [20]. The aim of our study was to demonstrate that a multimarker strategy could be used for risk prediction in these patients. For that purpose we used an “unbiased” proteomic technique for plasma profiling. The SELDI-TOF-MS technique has previously provided meaningful prognostic information in various diseases such as cancer [21,22]. For instance, Belluco et al. [23] reported a profile combining 7 ion m/z peaks that yielded a sensitive and specific diagnostic procedure to discriminate women with stage 1 breast cancer from women without breast cancer. To the best of our knowledge, the present study is the first demonstration that a proteomic approach may improve risk stratification in HF patients.

We paid close attention to the phenotyping of the study populations. It should be underlined that the patients were well treated regarding their HF status with more than 90% receiving ACE inhibitors and betablockers, reflecting a modern practice in CHF. In addition, all the main predictors of cardiovascular mortality (age, sex, aetiology of HF, NYHA class, LVEF, BNP, creatinine and peak VO_2_), previously identified in the literature [4,5,6,7], were assessed with few missing values.

The proteomic scores were built using plasma proteomic biomarker-classifier based on 42 ion m/z peaks differentially intense in the discovery cohort; this strategy of discriminatory patterns for disease detection has already been successful in previous studies [24]. To select a good biomarker panel and in order to minimize the effects of overfitting [25], we used 3 different statistical regression methods to set up proteomic scores. Our data in the validation population show that the proteomic scores provide information that are independent from the currently “classic” prognostic markers (NYHA class, LVEF, BNP, peak VO_2_). Incremental improvement in model performance with the proteomic scores was also demonstrated.

Our results thus suggest that a multimarker strategy based on plasma proteomics has the potential to be clinically useful for the risk stratification in HF patients. In practice however, it should be acknowledged that the SELDI-TOF-MS technique has some limitations including difficulties in ion *m/z* peak identification and/or low rate of new analytes going from discovery to accurate measurement by specific assays in routine clinical practice. In addition, pre-analytic treatment of the samples is time-consuming and this technology will not be commercially available in the next years. Nevertheless it should also be emphasized that emerging proteomic technologies may provide easier access to the global information contained in patient plasma proteome in the next future [26,27]. These technologies associated with advances in the field of computation biology employing artificial neural networks to analyze complex changes in multiple biomarkers simultaneously may potentially modify prognostic evaluation of HF patients [28].

## Supporting Information

S1 Methods Supplemental methods: proteomic analysis. (DOC)

S2 Methods Supplemental methods: statistical analysis. (DOC)

S1 Table List of ion *m/z* peaks detected in the discovery population. Each ion *m/z* peak detected is named using the initial “p” followed by its *m/z* value, the type of array on which it was detected (H50 or CM10) and then the laser intensity: low mass (LM) or high mass (HM). Ion *m/z* peak intensities are expressed as mean ± SD. The 42 ion *m/z* peaks that reach a significant p value after Bonferroni correction in the discovery population are highlighted in blue and were used to calculate the proteomic scores.
(DOC)

S2 Table Pearson correlation matrix of the 42 ion m/z peaks used to build the proteomic scores. Each ion peak detected is named using the initial “p” followed by its m/z value, the type of array on which it was detected (H50 or CM10) and then the laser intensity: low mass (LM) or high mass (HM).
(DOC)

S3 Table Pearson correlation matrix of the proteomic scores in the discovery population. The proteomic scores were developed using the support vector machine (SVM), the sparse partial least square discriminant analysis (sPLS-DA) and the lasso logistic regression (LASSO).
(DOC)

S1 Fig Representatitive mass spectra. **The upper mass spectrum was obtained from the H50 proteinchip array using low-mass (LM) parameter settings and the lower mass spectrum was obtained from the CM10 proteinchip array using high-mass (HM) parameter settings**.
(TIF)

## References

[1] Hoes AW, Mosterd A, Grobbee DE. An epidemic of heart failure? Recent evidence from Europe. Eur Heart J. 1998;19 Suppl L: L2–9. 9821002

[2] Mosterd A, Hoes AW. Clinical epidemiology of heart failure. Heart 2007;93: 1137–1146. 17699180 10.1136/hrt.2003.025270 PMC1955040

[3] Jhund PS, Macintyre K, Simpson CR, Lewsey JD, Stewart S, Redpath A, et al Long-term trends in first hospitalization for heart failure and subsequent survival between 1986 and 2003: a population study of 5.1 million people. Circulation 2009;119: 515–523. 10.1161/CIRCULATIONAHA.108.812172 19153268

[4] Cintron G, Johnson G, Francis G, Cobb F, Cohn JN. Prognostic significance of serial changes in left ventricular ejection fraction in patients with congestive heart failure. The V-HeFT VA Cooperative Studies Group. Circulation 1993;87: VI17–23. 8500235

[5] Hillege HL, Girbes AR, de Kam PJ, Boomsma F, de Zeeuw D, Charlesworth A, et al Renal function, neurohormonal activation, and survival in patients with chronic heart failure. Circulation 2000;102: 203–210. 10889132 10.1161/01.cir.102.2.203

[6] de Groote P, Dagorn J, Soudan B, Lamblin N, McFadden E, Bauters C. B-type natriuretic peptide and peak exercise oxygen consumption provide independent information for risk stratification in patients with stable congestive heart failure. J Am Coll Cardiol. 2004;43: 1584–1589. 15120815 10.1016/j.jacc.2003.11.059

[7] Rahimi K, Bennett D, Conrad N, Williams TM, Basu J, Dwight J, et al Risk prediction in patients with heart failure: a systematic review and analysis. JACC Heart Fail. 2014;2: 440–446. 10.1016/j.jchf.2014.04.008 25194291

[8] Ouwerkerk W, Voors AA, Zwinderman AH. Factors Influencing the Predictive Power of Models for Predicting Mortality and/or Heart Failure Hospitalization in Patients With Heart Failure. JACC Heart Fail. 2014;2: 429–436. 10.1016/j.jchf.2014.04.006 25194294

[9] Dubois E, Fertin M, Burdese J, Amouyel P, Bauters C, Pinet F. Cardiovascular proteomics: translational studies to develop novel biomarkers in heart failure and left ventricular remodeling. Proteomics Clin Appl. 2011;5: 57–66. 10.1002/prca.201000056 21246740

[10] Motiwala SR, Sarma A, Januzzi JL, O'Donoghue ML. Biomarkers in ACS and heart failure: should men and women be interpreted differently? Clin Chem. 2014;60: 35–43. 10.1373/clinchem.2013.202531 24255075

[11] Fertin M, Beseme O, Duban S, Amouyel P, Bauters C, Pinet F. Deep plasma proteomic analysis of patients with left ventricular remodeling after a first myocardial infarction. Proteomics Clin Appl. 2010;4: 654–673. 10.1002/prca.200900178 21137084

[12] De Groote P, Lamblin N, Mouquet F, Plichon D, McFadden E, Van Belle E, et al Impact of diabetes mellitus on long-term survival in patients with congestive heart failure. Eur Heart J. 2004;25: 656–662. 15084370 10.1016/j.ehj.2004.01.010

[13] de Groote P, Fertin M, Goeminne C, Petyt G, Peyrot S, Foucher-Hossein C, et al Right ventricular systolic function for risk stratification in patients with stable left ventricular systolic dysfunction: comparison of radionuclide angiography to echoDoppler parameters. Eur Heart J. 2012;33: 2672–2679. 10.1093/eurheartj/ehs080 22453651

[14] de Groote P, Fertin M, Duva Pentiah A, Goeminne C, Lamblin N, Bauters C. Long-Term Functional and Clinical Follow-Up of Patients With Heart Failure With Recovered Left Ventricular Ejection Fraction After beta-Blocker Therapy. Circ Heart Fail. 2014;7: 434–439. 10.1161/CIRCHEARTFAILURE.113.000813 24563449

[15] Guerrier L, Thulasiraman V, Castagna A, Fortis F, Lin S, Lomas L, et al Reducing protein concentration range of biological samples using solid-phase ligand libraries. J Chromatogr B Analyt Technol Biomed Life Sci. 2006;833: 33–40. 16455314 10.1016/j.jchromb.2005.12.048

[16] Thulasiraman V, Lin S, Gheorghiu L, Lathrop J, Lomas L, Hammond D, et al Reduction of the concentration difference of proteins in biological liquids using a library of combinatorial ligands. Electrophoresis 2005;26: 3561–3571. 16167368 10.1002/elps.200500147

[17] Karatzoglou A, Smola A, Hornik K, Zeileis A. kernlab—An S4 Package for Kernel Methods in R. J Stat Softw. 2004;11: 1–20.

[18] Chung D, Chun H, Keles S. spls: Sparse Partial Least Squares (SPLS) Regression and Classification. 2013; Available: http://CRANR-projectorg/package=spls.

[19] Friedman J, Hastie T, Tibshirani R. Regularization Paths for Generalized Linear Models via Coordinate Descent. J Stat Softw. 2010;33: 1–22. 20808728 PMC2929880

[20] McMurray JJ, Adamopoulos S, Anker SD, Auricchio A, Bohm M, Dickstein K, et al ESC Guidelines for the diagnosis and treatment of acute and chronic heart failure 2012: The Task Force for the Diagnosis and Treatment of Acute and Chronic Heart Failure 2012 of the European Society of Cardiology. Developed in collaboration with the Heart Failure Association (HFA) of the ESC. Eur Heart J. 2012;33: 1787–1847. 10.1093/eurheartj/ehs104 22611136

[21] Petrik V, Saadoun S, Loosemore A, Hobbs J, Opstad KS, Sheldon J, et al Serum alpha 2-HS glycoprotein predicts survival in patients with glioblastoma. Clin Chem. 2008;54: 713–722. 10.1373/clinchem.2007.096792 18281421

[22] Vermaat JS, van der Tweel I, Mehra N, Sleijfer S, Haanen JB, Roodhardt JM, et al Two-protein signature of novel serological markers apolipoprotein-A2 and serum amyloid alpha predicts prognosis in patients with metastatic renal cell cancer and improves the currently used prognostic survival models. Ann Oncol. 2010;21: 1472–1481. 10.1093/annonc/mdp559 20022911

[23] Belluco C, Petricoin EF, Mammano E, Facchiano F, Ross-Rucker S, Nitti D, et al Serum proteomic analysis identifies a highly sensitive and specific discriminatory pattern in stage 1 breast cancer. Ann Surg Oncol. 2007;14: 2470–2476. 17594124 10.1245/s10434-007-9354-3

[24] Nkuipou-Kenfack E, Duranton F, Gayrard N, Argiles A, Lundin U, Weinberger KM, et al Assessment of metabolomic and proteomic biomarkers in detection and prognosis of progression of renal function in chronic kidney disease. PLoS One 2014;9: e96955 10.1371/journal.pone.0096955 24817014 PMC4016198

[25] Hernandez B, Parnell A, Pennington SR. Why have so few proteomic biomarkers "survived" validation? (Sample size and independent validation considerations). Proteomics 2014;14: 1587–1592. 10.1002/pmic.201300377 24737731

[26] Boja ES, Rodriguez H. Mass spectrometry-based targeted quantitative proteomics: achieving sensitive and reproducible detection of proteins. Proteomics 2012;12: 1093–1110. 10.1002/pmic.201100387 22577011

[27] Peterman S, Niederkofler EE, Phillips DA, Krastins B, Kiernan UA, Tubbs KA, et al An automated, high-throughput method for targeted quantification of intact insulin and its therapeutic analogs in human serum or plasma coupling mass spectrometric immunoassay with high resolution and accurate mass detection (MSIA-HRAM). Proteomics 2014;14: 1445–1456. 10.1002/pmic.201300300 24668948

[28] Myers J, de Souza CR, Borghi-Silva A, Guazzi M, Chase P, Bensimhon D, et al A neural network approach to predicting outcomes in heart failure using cardiopulmonary exercise testing. Int J Cardiol. 2014;171: 265–269. 10.1016/j.ijcard.2013.12.031 24387896

