# Supplementary material for: Multimarker Proteomic Profiling for the Prediction of Cardiovascular Mortality in Patients with Chronic Heart Failure
Source: PLoS One. 2015 Apr 23;10(4):e0119265. doi: 10.1371/journal.pone.0119265 (PMC4408082; doi:10.1371/journal.pone.0119265)
Supplement: S1 Methods — (DOC) [file pone.0119265.s001.doc]

# Text S1. Supplemental methods: proteomic analysis

*Access to the deep proteome*

The combinatorial peptide ligand library (CPLL) method has been applied to each plasma sample with the ProteoMiner protein enrichment kit (Bio-Rad Laboratories, Hercules, CA, USA). After removing the storage solution (20% beads, 20% v/v aqueous EtOH, 0.5% v/v acetonitrile (ACN)) by centrifugation at 1,000 g for 2 min, the beads were washed with 1 mL deionized water followed by a 1 mL wash buffer (PBS buffer, 150 mM NaCl, 10 mM NaH_2_PO_4_, pH 7.4). In total, 1 mL of plasma sample was then added to columns containing 500 μL of washed beads and rotated end-to-end for 2 h at room temperature. Columns were then centrifuged to eliminate unbound proteins. Beads were then washed three times with 1 mL wash buffer and once with 1 mL deionized H_2_O. A total of 100-μL elution reagent (610 μL of 5% acid acetic in lyophilized elution reagent constituted of 4 mol/L urea, 1% CHAPS) was then added to each column at room temperature over a period of 15 min and elution was performed by centrifugation; entire step was repeated twice. Elution samples obtained from the three centrifugations were mixed together and divided into five aliquots of 50 μL and stored at -20°C.

*Proteomic analyses*

Arrays were prepared as follows: each sample (5 μL of CPLL-treated plasma) was diluted ten-fold in binding buffer, which varied according to array: for CM10, sodium acetate 100 mmol/L pH 4 and for H50, ACN 10%, trifluoroacid 0.1% and NaCl 150 mmol/L. The CM10 and H50 arrays were pre-equilibrated for 5 min in 100 μL binding buffer before sample addition. Each sample (50 μL) was incubated for 30 min in binding buffer. Samples were tested in duplicate and randomly distributed on arrays. For CM10 and H50 arrays, each spot was then washed twice for 5 min with the binding buffer and for 5 seconds with HEPES 5 mmol/L buffer. Then spots were allowed to dry partially before 2×0.6 μL of sinapinic acid was added. Finally, the spots were allowed to dry completely before SELDI-TOF-MS analysis.

To ensure sufficient coverage of the entire mass range, the acquisition settings for low mass (0–30000 Da) and high mass (>30000 Da) proteins were optimized separately. Arrays were analyzed with an ion acceleration potential of 25 kV. The mass range investigated was from 0 to 200 000 Da. Ten laser shots per pixel were averaged; two warning shots per pixel (data shot energy **+**10%) were taken before data collection but the warning shot data were not included in the data averages. The sampling rate was 400 MHz. Focus mass was 16000 Da for low mass and 40000 Da for high mass proteins. Matrix was attenuated below 2500 Da for low mass and below 10000 Da for high mass proteins. This set of acquisition parameters was combined with optimized laser energy determined by testing acquisition protocols on a reference and an experimental sample. Laser energies for low/high mass proteins respectively were: 2500/3500 nJ for CM10 arrays and 2200/2800 nJ for H50 arrays. Before each SELDI-TOF-MS analysis, a calibration step was performed. The calibrating mixture was composed of hirudin (6964 Da), cytochrome c (12230 Da), myoglobin (16951 Da), carbonic anhydrase (29023 Da), enolase (46671 Da), albumin (6433) and IgG (147300 Da). The calibration spot analysis used the same parameters as the sample analysis.

All data were processed with ProteinChip Data Manager software. Spectra were calibrated according to the calibration equation calculated the same day as the SELDI-TOF-MS analysis of one array type. Noise was calculated and spectra were normalized by total ion current. The analysis used spectra with normalization factor **<**3. Groups of ion *m/z* peaks of similar mass across spectra were assembled into clusters, according to two-step parameter settings. For the first step, ion *m/z* peaks were automatically detected according to the specified S/N (4 or 5) and the minimum valley depth (3 or 4), if they were found in at least 10% of all spectra, with an *m/z* error of less than 0.2%. Settings for the second step were a S/N of 2 and a minimum valley depth of 2. The *m/z* range was set between 2500 and 30 000 for low mass and between 30000 and 150000 for the high mass proteins. To monitor the quality of the assay, the coefficient of variation (CV) for spectra ion *m/z* peak intensity, derived from a single reference sample run every 2 arrays were calculated for each type of proteinchip array. Acquisition was performed with the same parameters as for the experimental sample. The ion *m/z* peak detection settings used with the experimental samples were also used for the reference samples. The CV for intensity was defined as the ratio of its standard deviation (SD) to its average and was calculated for each cluster. A pooled CV was calculated for each type of array and for low and high mass proteins, defined as the ratio of the sum of the squared CV of intensity of each ion *m/z* peak to the number of peaks. Finally, in order evaluate quality of spectra, we systematically calculate the normalization factor for each spectrum: any spectrum with a normalization factor greater than 3 and lower than 0.5 were removed. Spectra with normalization factor between either 0.5 and 1 or 2.5 and 3 were compared with their duplicates: if the two were similar, both were kept for final analysis; if not, the spectrum with the borderline normalization factor was removed. In case of spectrum exclusions after normalization, a novel normalization step was carried out.

# 
