# Supplementary material for: Multimarker Proteomic Profiling for the Prediction of Cardiovascular Mortality in Patients with Chronic Heart Failure
Source: PLoS One. 2015 Apr 23;10(4):e0119265. doi: 10.1371/journal.pone.0119265 (PMC4408082; doi:10.1371/journal.pone.0119265)
Supplement: S2 Methods — (DOC) [file pone.0119265.s002.doc]

# Text S2. Supplemental methods: statistical analysis

### Statistical analyses

General considerations: High throughput and non a-priori technologies such as the SELDI-TOF-MS technique usually allow measuring hundreds or thousands variables (ion peaks, proteins or protein fragments) simultaneously. The datasets, commonly called proteomic data, generated by these techniques are generally very large in terms of number of parameters (p, p=203 in the present study) and relatively small in terms of number of biological samples or patients (n, n=198 in the present study). In statistics, this problem is termed as the “large p and small n” problem (p>n). In such wide datasets and as it was the case in our study, there is often high colinearity between variables due to: p>n but also and primarily because of high correlation between variables related to common biological functions (proteins involved in the same physiological or pathophysiological pathways) or technical limits. In classical statistical methods, multiple logistic regression methods are used for prediction situations; but due to the high colinearity, these methods cannot be applied here. Therefore, we used different approaches: penalization regression methods or machine learning methods (SVM, sPLS-DA and LASSO) that are specifically dedicated to deal with such issues.

Development of the proteomic scores: The scores were derived from the proteomic data of the discovery population in 2 successive steps. First, we reduced the number of candidate ion *m/z* peaks (n = 203) using bivariate analysis. The mean intensity of each ion *m/z* peak was compared between cases and controls using a paired Student t-test. We then applied a Bonferroni correction to account for multiple testing, and statistical significance was thus defined as p-value <0.00025. Using this approach, we selected 42 significant ion *m/z* peaks for subsequent analyses. Correlation between these 42 ion *m/z* peaks was explored face-to-face using the Pearson correlation coefficient. In the second step, 3 different statistical regression methods were applied on the selected ion *m/z* peaks to discriminate cases and controls: the support vector machine method (SVM), the sparse partial least square discriminant analysis method (sPLS-DA), and a lasso logistic regression (LASSO). Each method contained specific “tuning” parameters that were chosen using re-sampling techniques. “SVM” is a kernel based method. The radial basis function kernel was used and the kernel parameter was estimated analytically with a cost parameter ranging from 0.1 to 1000. “sPLS-DA” is a variant of the partial least square (PLS) method where the response is categorical. This method admitted 2 “tuning” parameters: the number of components (*k*) ranging from 1 to 42 and the sparsity (*eta*) ranging from 0.01 to 0.99. “LASSO” is a penalized regression model. Penalization was reflected by the “tuning” parameter *lambda* ranging from 0.01 to 0.99. We used the following R packages: “kernlab” R package (version 0.9-19) for SVM [1], “spls” R package (version 2.2-1) for sPLS-DA [2] and “glmnet” R package (version 1.9-5) for LASSO [3]. For each method, we determined the parameters maximizing the area under the curve (AUC) using receiver operating characteristic (ROC) curve analysis and the model parsimony by running 10 cross-validations repeated 5 times with the “train” function included in the “caret” R package (version 5.17-7) [4]. Because of high colinearity between the 42 selected ion *m/z* peaks, cross-validations resulted in a set of parameters reflecting a high penalization, a high sparsity or a low complexity (cost=2 for SVM, eta=0.98 for sPLS-DA based on 15 hidden components and lambda=0.01 for LASSO). Proteomic scores were predicted values obtained with the 3 models. Comparisons of the proteomic score values between cases and controls were performed using paired Student’s t-test.

Validation of the proteomic scores: The 3 models were applied in the validation population to compute the predicted probabilities of cardiovascular death. Comparisons of the proteomic score values between patients who experienced cardiovascular death and those who were still alive at the end of follow-up in the validation population were performed using unpaired Student’s t-test. We evaluate the discrimination performance of the proteomic scores to predict cardiovascular death by means of AUC using ROC curve analyses.

All statistical analyses were performed using R Statistical Package version 3.0.

#### References
