# Supplementary material for: Multimarker Proteomic Profiling for the Prediction of Cardiovascular Mortality in Patients with Chronic Heart Failure
Source: PLoS One. 2015 Apr 23;10(4):e0119265. doi: 10.1371/journal.pone.0119265 (PMC4408082; doi:10.1371/journal.pone.0119265)
Supplement: S1 Table — Each ion m/z peak detected is named using the initial “p” followed by its m/z value, the type of array on which it was detected (H50 or CM10) and then the laser intensity: low mass (LM) or high mass (HM). Ion m/z peak intensities are expressed as mean ± SD. The 42 ion m/z peaks that reach a significant p value after Bonferroni correction in the discovery population are highlighted in blue and were used to calculate the proteomic scores. (DOC) [file pone.0119265.s003.doc]

**Table S1. List of ion *m/z* peaks detected in the discovery population.**

|  | **Controls**  **(n=99)** | **Cases**  **(n=99)** | **P value** |
| --- | --- | --- | --- |
| p2816-H50/LM | 7.68 ± 2.74 | 7.54 ± 2.27 | 7.07E-01 |
| p3166-H50/LM | 3.36 ± 1.13 | 3.92 ± 1.35 | 1.22E-03 |
| p3267-H50/LM | 5.03 ± 1.89 | 6.51 ± 2.39 | 4.15E-06 |
| p4430-H50/LM | 3.19 ± 1.10 | 3.48 ± 1.24 | 9.58E-02 |
| p4679-H50/LM | 2.65 ± 1.34 | 3.20 ± 1.24 | 2.97E-03 |
| p6416-H50/LM | 34.72 ± 19.64 | 48.23 ± 23.37 | 3.96E-05 |
| p6616-H50/LM | 70.28 ± 37.53 | 99.15 ± 43.67 | 3.62E-06 |
| p6825-H50/LM | 6.24 ± 3.12 | 8.65 ± 3.65 | 3.93E-06 |
| p7147-H50/LM | 1.52 ± 1.54 | 4.40 ± 7.70 | 3.12E-04 |
| p7681-H50/LM | 1.47 ± 0.75 | 2.24 ± 1.09 | 4.44E-08 |
| p7781-H50/LM | 1.48 ± 1.17 | 2.37 ± 2.54 | 7.22E-04 |
| p8206-H50/LM | 3.82 ± 3.33 | 4.82 ± 3.59 | 2.96E-02 |
| p8228-H50/LM | 2.83 ± 2.54 | 2.54 ± 2.60 | 3.88E-01 |
| p8568-H50/LM | 2.81 ± 1.99 | 2.74 ± 1.52 | 7.68E-01 |
| p8692-H50/LM | 9.44 ± 3.89 | 10.75 ± 5.24 | 5.14E-02 |
| p8764-H50/LM | 11.73 ± 10.88 | 16.07 ± 12.34 | 1.47E-02 |
| p8813-H50/LM | 10.59 ± 5.43 | 12.35 ± 6.24 | 4.23E-02 |
| p8910-H50/LM | 25.56 ± 16.30 | 33.78 ± 19.70 | 1.95E-03 |
| p8939-H50/LM | 13.96 ± 8.67 | 13.26 ± 7.48 | 5.23E-01 |
| p9136-H50/LM | 7.55 ± 4.56 | 9.66 ± 4.69 | 1.97E-03 |
| p9361-H50/LM | 5.85 ± 1.52 | 6.10 ± 1.83 | 2.94E-01 |
| p9421-H50/LM | 16.42 ± 14.55 | 23.46 ± 15.98 | 1.29E-03 |
| p9712-H50/LM | 4.08 ± 2.98 | 5.42 ± 2.91 | 8.38E-04 |
| p10436-H50/LM | 1.09 ± 1.59 | 2.04 ± 3.99 | 2.92E-02 |
| p11447-H50/LM | 1.14 ± 2.72 | 1.39 ± 3.25 | 5.72E-01 |
| p11532-H50/LM | 3.69 ± 9.20 | 4.56 ± 10.23 | 5.39E-01 |
| p11552-H50/LM | 2.19 ± 3.69 | 2.63 ± 6.09 | 5.43E-01 |
| p11688-H50/LM | 3.64 ± 5.19 | 6.37 ± 16.28 | 1.17E-01 |
| p11701-H50/LM | 2.81 ± 3.85 | 4.59 ± 12.55 | 1.84E-01 |
| p12589-H50/LM | 1.99 ± 1.01 | 1.89 ± 1.42 | 5.43E-01 |
| p12869-H50/LM | 4.46 ± 1.90 | 4.48 ± 2.19 | 9.45E-01 |
| p12887-H50/LM | 4.89 ± 2.59 | 4.24 ± 3.52 | 8.05E-02 |
| p14060-H50/LM | 15.08 ± 5.10 | 11.96 ± 4.77 | 7.51E-05 |
| p14166-H50/LM | 6.67 ± 2.14 | 5.30 ± 2.02 | 3.74E-05 |
| p14515-H50/LM | 2.57 ± 0.86 | 2.03 ± 0.76 | 2.70E-05 |
| p14813-H50/LM | 1.32 ± 0.38 | 1.37 ± 1.28 | 7.20E-01 |
| p15743-H50/LM | 0.34 ± 0.22 | 0.49 ± 0.44 | 1.52E-03 |
| p15869-H50/LM | 0.51 ± 0.35 | 0.76 ± 0.61 | 2.78E-04 |
| p16095-H50/LM | 0.62 ± 0.49 | 0.80 ± 0.58 | 1.29E-02 |
| p16249-H50/LM | 1.07 ± 0.70 | 1.35 ± 0.84 | 3.01E-03 |
| p16382-H50/LM | 0.61 ± 0.47 | 0.77 ± 0.53 | 1.12E-02 |
| p17156-H50/LM | 1.62 ± 0.98 | 1.52 ± 0.94 | 4.65E-01 |
| p17286-H50/LM | 3.72 ± 1.67 | 3.66 ± 1.88 | 7.88E-01 |
| p17412-H50/LM | 4.08 ± 1.94 | 3.96 ± 2.12 | 6.85E-01 |
| p17619-H50/LM | 1.29 ± 0.59 | 1.24 ± 0.61 | 6.13E-01 |
| p21766-H50/LM | 0.44 ± 0.24 | 0.50 ± 0.25 | 8.65E-02 |
| p22722-H50/LM | 0.22 ± 0.06 | 0.22 ± 0.07 | 8.31E-01 |
| p25898-H50/LM | 0.43 ± 0.31 | 0.55 ± 0.65 | 9.03E-02 |

|  | **Controls**  **(n=99)** | **Cases**  **(n=99)** | **P value** |
| --- | --- | --- | --- |
| p26022-H50/LM | 0.43 ± 0.28 | 0.50 ± 0.46 | 1.60E-01 |
| p28134-H50/LM | 18.76 ± 6.07 | 14.46 ± 5.86 | 6.05E-06 |
| p28303-H50/LM | 10.17 ± 3.23 | 7.84 ± 3.10 | 4.05E-06 |
| p29027-H50/LM | 4.51 ± 1.55 | 3.26 ± 1.26 | 5.89E-08 |
| p100299-H50/HM | 0.11 ± 0.04 | 0.12 ± 0.04 | 2.81E-03 |
| p102431-H50/HM | 0.07 ± 0.01 | 0.06 ± 0.02 | 6.56E-01 |
| p113729-H50/HM | 0.23 ± 0.06 | 0.17 ± 0.07 | 2.62E-08 |
| p129794-H50/HM | 0.04 ± 0.01 | 0.04 ± 0.01 | 7.64E-01 |
| p20475-H50/HM | 0.23 ± 0.09 | 0.32 ± 0.26 | 1.12E-03 |
| p21723-H50/HM | 0.97 ± 0.59 | 1.38 ± 0.71 | 7.83E-06 |
| p21816-H50/HM | 0.69 ± 0.37 | 0.97 ± 0.47 | 5.30E-06 |
| p22752-H50/HM | 0.56 ± 0.21 | 0.68 ± 0.21 | 2.63E-05 |
| p23922-H50/HM | 0.31 ± 0.14 | 0.46 ± 0.26 | 7.36E-07 |
| p24017-H50/HM | 0.34 ± 0.14 | 0.51 ± 0.32 | 6.17E-06 |
| p25672-H50/HM | 0.85 ± 0.27 | 1.27 ± 1.32 | 1.61E-03 |
| p25877-H50/HM | 1.44 ± 0.79 | 2.74 ± 4.51 | 4.04E-03 |
| p26026-H50/HM | 1.39 ± 0.64 | 2.19 ± 2.44 | 1.04E-03 |
| p26610-H50/HM | 1.47 ± 0.48 | 1.92 ± 1.32 | 7.62E-04 |
| p26777-H50/HM | 1.54 ± 0.46 | 1.96 ± 1.36 | 1.64E-03 |
| p28124-H50/HM | 79.86 ± 7.99 | 78.04 ± 10.96 | 2.21E-01 |
| p28328-H50/HM | 32.71 ± 2.50 | 31.48 ± 3.96 | 1.48E-02 |
| p28523-H50/HM | 12.93 ± 0.96 | 12.24 ± 1.57 | 2.12E-04 |
| p28992-H50/HM | 13.08 ± 1.56 | 11.66 ± 1.52 | 2.22E-09 |
| p29188-H50/HM | 9.59 ± 0.84 | 8.76 ± 1.01 | 2.38E-09 |
| p34415-H50/HM | 0.72 ± 0.40 | 0.89 ± 0.83 | 6.35E-02 |
| p34594-H50/HM | 0.78 ± 0.36 | 0.98 ± 0.58 | 7.29E-03 |
| p34801-H50/HM | 0.85 ± 0.39 | 1.08 ± 0.54 | 1.16E-03 |
| p35840-H50/HM | 0.40 ± 0.16 | 0.53 ± 0.20 | 5.72E-06 |
| p36972-H50/HM | 0.67 ± 0.44 | 0.84 ± 0.56 | 2.53E-02 |
| p37561-H50/HM | 0.49 ± 0.34 | 0.65 ± 0.46 | 6.96E-03 |
| p39750-H50/HM | 0.13 ± 0.22 | 0.21 ± 0.48 | 1.45E-01 |
| p41132-H50/HM | 0.29 ± 0.11 | 0.31 ± 0.15 | 1.59E-01 |
| p43483-H50/HM | 2.41 ± 1.53 | 3.35 ± 1.69 | 1.65E-05 |
| p45495-H50/HM | 0.74 ± 0.29 | 0.82 ± 0.32 | 5.83E-02 |
| p54233-H50/HM | 0.37 ± 0.12 | 0.43 ± 0.24 | 9.28E-03 |
| p56387-H50/HM | 3.89 ± 0.49 | 3.45 ± 0.75 | 5.94E-06 |
| p57201-H50/HM | 2.00 ± 0.34 | 1.64 ± 0.40 | 3.05E-10 |
| p62947-H50/HM | 0.15 ± 0.07 | 0.17 ± 0.10 | 1.22E-01 |
| p65380-H50/HM | 0.16 ± 0.08 | 0.17 ± 0.08 | 1.87E-01 |
| p69237-H50/HM | 0.09 ± 0.04 | 0.11 ± 0.09 | 2.91E-02 |
| p71729-H50/HM | 0.43 ± 0.19 | 0.54 ± 0.19 | 1.26E-05 |
| p73852-H50/HM | 0.21 ± 0.06 | 0.22 ± 0.06 | 2.13E-02 |
| p84946-H50/HM | 0.79 ± 0.16 | 0.64 ± 0.21 | 1.95E-07 |
| p91443-H50/HM | 0.04 ± 0.02 | 0.04 ± 0.02 | 7.05E-01 |
| p94005-H50/HM | 0.03 ± 0.02 | 0.03 ± 0.02 | 8.54E-01 |
| p142405-H50/HM | 0.08 ± 0.03 | 0.06 ± 0.03 | 9.25E-08 |
| p2696-CM10/LM | 11.67 ± 7.43 | 11.46 ± 4.97 | 7.87E-01 |
| p2817-CM10/LM | 8.48 ± 2.63 | 7.87 ± 2.44 | 9.35E-02 |
| p2884-CM10/LM | 5.36 ± 3.05 | 5.42 ± 2.37 | 8.56E-01 |
| p3167-CM10/LM | 3.98 ± 1.07 | 4.21 ± 1.22 | 1.46E-01 |

|  | **Controls**  **(n=99)** | **Cases**  **(n=99)** | **P value** |
| --- | --- | --- | --- |
| p3268-CM10/LM | 7.48 ± 1.86 | 7.90 ± 2.01 | 1.18E-01 |
| p3847-CM10/LM | 2.03 ± 0.96 | 1.87 ± 0.93 | 2.54E-01 |
| p4431-CM10/LM | 1.71 ± 0.66 | 1.51 ± 0.75 | 3.58E-02 |
| p5052-CM10/LM | 1.66 ± 0.97 | 1.54 ± 0.85 | 2.75E-01 |
| p5181-CM10/LM | 0.93 ± 0.94 | 1.41 ± 1.48 | 2.65E-03 |
| p5885-CM10/LM | 1.15 ± 1.09 | 1.11 ± 1.03 | 7.95E-01 |
| p6420-CM10/LM | 69.40 ± 24.47 | 73.42 ± 26.33 | 2.53E-01 |
| p6620-CM10/LM | 153.61 ± 46.91 | 162.57 ± 45.34 | 1.58E-01 |
| p6828-CM10/LM | 14.88 ± 4.72 | 15.80 ± 4.20 | 1.53E-01 |
| p7001-CM10/LM | 2.33 ± 0.90 | 2.69 ± 1.29 | 2.27E-02 |
| p7149-CM10/LM | 1.22 ± 0.72 | 1.93 ± 1.83 | 3.45E-04 |
| p7752-CM10/LM | 17.29 ± 16.12 | 10.98 ± 12.18 | 3.40E-03 |
| p7925-CM10/LM | 2.36 ± 1.50 | 1.90 ± 1.39 | 3.73E-02 |
| p8133-CM10/LM | 3.05 ± 2.79 | 2.37 ± 2.36 | 7.15E-02 |
| p8276-CM10/LM | 1.84 ± 1.08 | 1.95 ± 1.29 | 5.14E-01 |
| p8593-CM10/LM | 3.97 ± 2.39 | 5.38 ± 3.69 | 9.02E-04 |
| p8645-CM10/LM | 2.64 ± 1.34 | 3.55 ± 1.86 | 4.43E-05 |
| p8684-CM10/LM | 2.85 ± 0.81 | 2.92 ± 0.96 | 6.11E-01 |
| p8764-CM10/LM | 5.89 ± 3.85 | 6.62 ± 4.24 | 2.43E-01 |
| p8925-CM10/LM | 16.93 ± 10.43 | 14.25 ± 10.07 | 7.11E-02 |
| p9088-CM10/LM | 16.80 ± 11.11 | 11.80 ± 8.80 | 9.09E-04 |
| p9132-CM10/LM | 4.16 ± 1.90 | 3.97 ± 1.49 | 4.33E-01 |
| p9308-CM10/LM | 5.18 ± 3.14 | 3.77 ± 1.97 | 3.31E-04 |
| p9430-CM10/LM | 6.27 ± 3.67 | 8.36 ± 6.83 | 8.46E-03 |
| p9631-CM10/LM | 1.93 ± 0.85 | 2.35 ± 1.07 | 7.74E-04 |
| p9930-CM10/LM | 1.16 ± 0.59 | 1.65 ± 1.31 | 8.57E-04 |
| p10083-CM10/LM | 3.58 ± 2.65 | 2.60 ± 1.67 | 2.61E-03 |
| p10274-CM10/LM | 1.76 ± 1.55 | 2.21 ± 2.17 | 6.45E-02 |
| p10305-CM10/LM | 1.93 ± 2.13 | 2.79 ± 3.22 | 2.54E-02 |
| p10398-CM10/LM | 3.96 ± 5.42 | 6.68 ± 11.39 | 2.44E-02 |
| p10429-CM10/LM | 6.10 ± 8.22 | 10.77 ± 14.24 | 1.57E-03 |
| p10647-CM10/LM | 1.35 ± 1.56 | 2.34 ± 2.87 | 9.55E-04 |
| p10827-CM10/LM | 5.26 ± 2.97 | 5.54 ± 2.92 | 5.30E-01 |
| p11378-CM10/LM | 0.78 ± 0.79 | 1.38 ± 2.26 | 1.02E-02 |
| p11541-CM10/LM | 1.54 ± 3.73 | 2.06 ± 5.47 | 4.42E-01 |
| p11686-CM10/LM | 1.87 ± 2.96 | 3.44 ± 10.38 | 1.52E-01 |
| p11707-CM10/LM | 1.49 ± 2.01 | 2.53 ± 7.93 | 2.10E-01 |
| p11899-CM10/LM | 0.68 ± 0.65 | 1.09 ± 1.65 | 2.66E-02 |
| p12377-CM10/LM | 0.52 ± 0.53 | 0.52 ± 0.47 | 9.65E-01 |
| p12601-CM10/LM | 0.75 ± 0.39 | 0.67 ± 0.40 | 1.54E-01 |
| p12770-CM10/LM | 1.43 ± 0.80 | 2.02 ± 1.13 | 1.60E-05 |
| p12900-CM10/LM | 1.97 ± 0.89 | 1.85 ± 0.69 | 2.12E-01 |
| p13081-CM10/LM | 1.47 ± 0.76 | 1.52 ± 0.81 | 6.41E-01 |
| p13282-CM10/LM | 1.16 ± 0.60 | 1.26 ± 0.57 | 2.30E-01 |
| p13582-CM10/LM | 0.97 ± 0.52 | 0.93 ± 0.58 | 5.86E-01 |
| p13806-CM10/LM | 1.34 ± 0.37 | 1.24 ± 0.39 | 6.86E-02 |
| p13854-CM10/LM | 1.25 ± 0.39 | 1.49 ± 0.56 | 1.16E-03 |
| p13895-CM10/LM | 2.08 ± 0.59 | 1.87 ± 0.59 | 1.32E-02 |
| p14064-CM10/LM | 5.97 ± 2.38 | 4.71 ± 1.81 | 1.75E-04 |
| p14151-CM10/LM | 3.52 ± 0.97 | 2.90 ± 0.83 | 5.95E-06 |

|  | **Controls**  **(n=99)** | **Cases**  **(n=99)** | **P value** |
| --- | --- | --- | --- |
| p14511-CM10/LM | 1.23 ± 0.45 | 0.89 ± 0.32 | 4.94E-08 |
| p14727-CM10/LM | 1.76 ± 0.66 | 1.63 ± 0.61 | 1.46E-01 |
| p15150-CM10/LM | 0.56 ± 0.53 | 0.34 ± 0.26 | 2.37E-04 |
| p15893-CM10/LM | 0.57 ± 1.63 | 0.40 ± 0.66 | 3.31E-01 |
| p16235-CM10/LM | 0.20 ± 0.20 | 0.27 ± 0.26 | 2.33E-02 |
| p17284-CM10/LM | 1.10 ± 0.32 | 1.05 ± 0.36 | 3.61E-01 |
| p17398-CM10/LM | 1.14 ± 0.52 | 1.05 ± 0.45 | 1.79E-01 |
| p19394-CM10/LM | 0.39 ± 0.49 | 0.71 ± 0.87 | 7.02E-04 |
| p19508-CM10/LM | 0.52 ± 0.61 | 0.91 ± 1.04 | 5.31E-04 |
| p20525-CM10/LM | 0.51 ± 0.44 | 0.91 ± 1.00 | 1.27E-04 |
| p21766-CM10/LM | 0.18 ± 0.09 | 0.21 ± 0.13 | 3.51E-02 |
| p22700-CM10/LM | 0.17 ± 0.05 | 0.17 ± 0.06 | 2.93E-01 |
| p28127-CM10/LM | 6.85 ± 2.97 | 5.26 ± 2.36 | 2.87E-04 |
| p28311-CM10/LM | 3.92 ± 1.65 | 3.03 ± 1.31 | 2.66E-04 |
| p29024-CM10/LM | 1.86 ± 0.84 | 1.32 ± 0.59 | 3.94E-06 |
| p20508-CM10/HM | 1.31 ± 1.19 | 2.62 ± 3.17 | 1.11E-04 |
| p21716-CM10/HM | 0.38 ± 0.16 | 0.48 ± 0.27 | 1.16E-03 |
| p22236-CM10/HM | 0.18 ± 0.12 | 0.20 ± 0.14 | 3.79E-01 |
| p22694-CM10/HM | 0.40 ± 0.13 | 0.46 ± 0.16 | 9.88E-03 |
| p23044-CM10/HM | 0.31 ± 0.23 | 0.35 ± 0.53 | 4.72E-01 |
| p25907-CM10/HM | 0.64 ± 0.49 | 0.92 ± 1.23 | 2.52E-02 |
| p26011-CM10/HM | 0.53 ± 0.36 | 0.69 ± 0.70 | 3.72E-02 |
| p26783-CM10/HM | 0.48 ± 0.27 | 0.54 ± 0.46 | 2.14E-01 |
| p28121-CM10/HM | 26.67 ± 6.37 | 23.01 ± 7.04 | 2.61E-04 |
| p28321-CM10/HM | 11.70 ± 2.51 | 10.08 ± 2.76 | 4.74E-05 |
| p28505-CM10/HM | 4.99 ± 1.07 | 4.22 ± 1.13 | 4.17E-06 |
| p29000-CM10/HM | 5.36 ± 1.32 | 4.17 ± 1.08 | 2.61E-10 |
| p29188-CM10/HM | 3.71 ± 0.84 | 2.97 ± 0.76 | 1.86E-09 |
| p33372-CM10/HM | 0.36 ± 0.18 | 0.41 ± 0.21 | 9.68E-02 |
| p34290-CM10/HM | 1.76 ± 0.95 | 1.76 ± 1.54 | 9.94E-01 |
| p34408-CM10/HM | 1.77 ± 0.79 | 1.70 ± 1.18 | 6.00E-01 |
| p37296-CM10/HM | 1.03 ± 0.71 | 1.21 ± 0.71 | 7.65E-02 |
| p37590-CM10/HM | 1.25 ± 0.88 | 1.49 ± 0.88 | 4.62E-02 |
| p37823-CM10/HM | 0.98 ± 0.64 | 1.20 ± 0.64 | 1.54E-02 |
| p39859-CM10/HM | 0.24 ± 0.17 | 0.33 ± 0.22 | 1.38E-03 |
| p43441-CM10/HM | 1.10 ± 0.44 | 1.34 ± 0.59 | 2.73E-04 |
| p50133-CM10/HM | 0.47 ± 0.19 | 0.52 ± 0.23 | 7.61E-02 |
| p51398-CM10/HM | 0.41 ± 0.25 | 0.36 ± 0.21 | 1.59E-01 |
| p54075-CM10/HM | 0.12 ± 0.07 | 0.13 ± 0.07 | 3.78E-01 |
| p56259-CM10/HM | 1.08 ± 0.40 | 0.84 ± 0.36 | 3.29E-05 |
| p59418-CM10/HM | 0.22 ± 0.08 | 0.24 ± 0.08 | 1.08E-01 |
| p62841-CM10/HM | 0.35 ± 0.10 | 0.38 ± 0.11 | 3.82E-02 |
| p63755-CM10/HM | 0.51 ± 0.31 | 0.59 ± 0.28 | 4.15E-02 |
| p66600-CM10/HM | 1.17 ± 0.66 | 1.28 ± 0.77 | 2.35E-01 |
| p68543-CM10/HM | 0.48 ± 0.20 | 0.48 ± 0.22 | 8.80E-01 |
| p71500-CM10/HM | 0.78 ± 0.20 | 0.82 ± 0.22 | 2.07E-01 |
| p78229-CM10/HM | 0.28 ± 0.11 | 0.34 ± 0.14 | 8.09E-04 |
| p84478-CM10/HM | 0.16 ± 0.08 | 0.12 ± 0.06 | 2.71E-03 |
| p89245-CM10/HM | 0.25 ± 0.12 | 0.35 ± 0.15 | 2.99E-06 |
| p94504-CM10/HM | 0.07 ± 0.03 | 0.07 ± 0.03 | 9.33E-01 |

|  | **Controls**  **(n=99)** | **Cases**  **(n=99)** | **P value** |
| --- | --- | --- | --- |
| p99364-CM10/HM | 0.10 ± 0.03 | 0.09 ± 0.03 | 1.72E-01 |
| p103419-CM10/HM | 0.03 ± 0.02 | 0.03 ± 0.03 | 9.34E-01 |
| p112830-CM10/HM | 0.04 ± 0.02 | 0.03 ± 0.01 | 6.00E-04 |
| p138821-CM10/HM | 0.04 ± 0.02 | 0.04 ± 0.02 | 2.23E-03 |
| p142197-CM10/HM | 0.04 ± 0.02 | 0.06 ± 0.03 | 2.12E-03 |

Each ion *m/z* peak detected is named using the initial “p” followed by its *m/z* value, the type of array on which it was detected (H50 or CM10) and then the laser intensity: low mass (LM) or high mass (HM). Ion *m/z* peak intensities are expressed as mean ± SD. The 42 ion *m/z* peaks that reach a significant p value after Bonferroni correction in the discovery population are highlighted in blue and were used to calculate the proteomic scores.
