# Supplementary material for: Multimarker Proteomic Profiling for the Prediction of Cardiovascular Mortality in Patients with Chronic Heart Failure
Source: PLoS One. 2015 Apr 23;10(4):e0119265. doi: 10.1371/journal.pone.0119265 (PMC4408082; doi:10.1371/journal.pone.0119265)
Supplement: S2 Table — Each ion peak detected is named using the initial “p” followed by its m/z value, the type of array on which it was detected (H50 or CM10) and then the laser intensity: low mass (LM) or high mass (HM). (DOC) [file pone.0119265.s004.doc]

Table S2. Pearson correlation matrix of the 42 ion m/z peaks used to build the proteomic scores.

|  | **p8645-CM10/LM** | **p12770-CM10/LM** | **p14064-CM10/LM** | **p14151-CM10/LM** | **p14511-CM10/LM** | **p15150-CM10/LM** |
| --- | --- | --- | --- | --- | --- | --- |
| **p142405-H50/HM** | -0.162 | -0.132 | 0.44 | 0.413 | 0.52 | 0.096 |
| **p113729-H50/HM** | -0.167 | -0.138 | 0.453 | 0.427 | 0.524 | 0.082 |
| **p84946-H50/HM** | -0.141 | -0.125 | 0.423 | 0.424 | 0.482 | 0.059 |
| **p71729-H50/HM** | 0.216 | 0.233 | -0.31 | -0.267 | -0.38 | -0.113 |
| **p57201-H50/HM** | -0.177 | -0.13 | 0.48 | 0.479 | 0.612 | 0.181 |
| **p56387-H50/HM** | -0.115 | -0.097 | 0.377 | 0.388 | 0.422 | 0.041 |
| **p43483-H50/HM** | 0.209 | 0.196 | -0.325 | -0.306 | -0.393 | -0.094 |
| **p35840-H50/HM** | 0.094 | 0.116 | -0.386 | -0.347 | -0.497 | -0.051 |
| **p29188-H50/HM** | -0.144 | -0.033 | 0.37 | 0.375 | 0.514 | 0.208 |
| **p28992-H50/HM** | -0.169 | -0.068 | 0.341 | 0.327 | 0.543 | 0.188 |
| **p28523-H50/HM** | 0.021 | 0.058 | 0.299 | 0.305 | 0.277 | 0.071 |
| **p24017-H50/HM** | 0.17 | 0.145 | -0.335 | -0.351 | -0.373 | -0.068 |
| **p23922-H50/HM** | 0.149 | 0.167 | -0.363 | -0.337 | -0.388 | -0.072 |
| **p22752-H50/HM** | 0.291 | 0.328 | -0.364 | -0.271 | -0.389 | -0.144 |
| **p21816-H50/HM** | 0.195 | 0.16 | -0.349 | -0.348 | -0.42 | -0.105 |
| **p21723-H50/HM** | 0.199 | 0.17 | -0.334 | -0.328 | -0.4 | -0.114 |
| **p29027-H50/LM** | -0.23 | -0.229 | 0.5 | 0.462 | 0.594 | 0.139 |
| **p28303-H50/LM** | -0.196 | -0.229 | 0.463 | 0.434 | 0.48 | 0.096 |
| **p28134-H50/LM** | -0.201 | -0.236 | 0.457 | 0.424 | 0.47 | 0.089 |
| **p14515-H50/LM** | -0.226 | -0.29 | 0.375 | 0.283 | 0.501 | 0.114 |
| **p14166-H50/LM** | -0.173 | -0.207 | 0.461 | 0.421 | 0.466 | 0.09 |
| **p14060-H50/LM** | -0.181 | -0.217 | 0.459 | 0.404 | 0.454 | 0.097 |
| **p7681-H50/LM** | 0.211 | 0.102 | -0.367 | -0.446 | -0.464 | -0.124 |
| **p6825-H50/LM** | 0.328 | 0.367 | -0.42 | -0.429 | -0.49 | -0.076 |
| **p6616-H50/LM** | 0.307 | 0.345 | -0.421 | -0.425 | -0.489 | -0.064 |
| **p6416-H50/LM** | 0.196 | 0.225 | -0.392 | -0.387 | -0.432 | -0.058 |
| **p3267-H50/LM** | 0.335 | 0.379 | -0.387 | -0.397 | -0.47 | -0.113 |
| **p89245-CM10/HM** | 0.391 | 0.444 | -0.526 | -0.343 | -0.507 | -0.101 |
| **p56259-CM10/HM** | -0.44 | -0.421 | 0.79 | 0.597 | 0.711 | 0.133 |
| **p29188-CM10/HM** | -0.414 | -0.387 | 0.733 | 0.558 | 0.777 | 0.14 |
| **p29000-CM10/HM** | -0.405 | -0.376 | 0.705 | 0.524 | 0.786 | 0.119 |
| **p28505-CM10/HM** | -0.41 | -0.397 | 0.735 | 0.572 | 0.677 | 0.156 |
| **p28321-CM10/HM** | -0.389 | -0.373 | 0.727 | 0.51 | 0.62 | 0.134 |
| **p20508-CM10/HM** | 0.23 | 0.078 | -0.34 | -0.471 | -0.395 | -0.158 |
| **p29024-CM10/LM** | -0.432 | -0.396 | 0.942 | 0.753 | 0.946 | 0.212 |
| **p20525-CM10/LM** | 0.192 | 0.047 | -0.239 | -0.4 | -0.294 | -0.135 |
| **p15150-CM10/LM** | -0.14 | -0.123 | 0.236 | 0.21 | 0.177 | 1 |
| **p14511-CM10/LM** | -0.374 | -0.326 | 0.882 | 0.797 | 1 | 0.177 |
| **p14151-CM10/LM** | -0.379 | -0.322 | 0.766 | 1 | 0.797 | 0.21 |
| **p14064-CM10/LM** | -0.4 | -0.363 | 1 | 0.766 | 0.882 | 0.236 |
| **p12770-CM10/LM** | 0.856 | 1 | -0.363 | -0.322 | -0.326 | -0.123 |
| **p8645-CM10/LM** | 1 | 0.856 | -0.4 | -0.379 | -0.374 | -0.14 |

|  | **p20525-CM10/LM** | **p29024-CM10/LM** | **p20508-CM10/HM** | **p28321-CM10/HM** | **p28505-CM10/HM** | **p29000-CM10/HM** |
| --- | --- | --- | --- | --- | --- | --- |
| **p142405-H50/HM** | -0.386 | 0.508 | -0.4 | 0.469 | 0.536 | 0.516 |
| **p113729-H50/HM** | -0.431 | 0.514 | -0.447 | 0.506 | 0.572 | 0.537 |
| **p84946-H50/HM** | -0.492 | 0.469 | -0.503 | 0.517 | 0.573 | 0.518 |
| **p71729-H50/HM** | 0.2 | -0.416 | 0.203 | -0.352 | -0.418 | -0.438 |
| **p57201-H50/HM** | -0.538 | 0.59 | -0.556 | 0.502 | 0.592 | 0.618 |
| **p56387-H50/HM** | -0.579 | 0.408 | -0.579 | 0.51 | 0.554 | 0.489 |
| **p43483-H50/HM** | 0.342 | -0.418 | 0.345 | -0.399 | -0.462 | -0.466 |
| **p35840-H50/HM** | 0.194 | -0.474 | 0.236 | -0.464 | -0.538 | -0.519 |
| **p29188-H50/HM** | -0.593 | 0.482 | -0.595 | 0.409 | 0.477 | 0.582 |
| **p28992-H50/HM** | -0.461 | 0.486 | -0.463 | 0.334 | 0.391 | 0.621 |
| **p28523-H50/HM** | -0.616 | 0.299 | -0.616 | 0.418 | 0.454 | 0.329 |
| **p24017-H50/HM** | 0.397 | -0.371 | 0.435 | -0.394 | -0.439 | -0.434 |
| **p23922-H50/HM** | 0.31 | -0.398 | 0.338 | -0.401 | -0.451 | -0.431 |
| **p22752-H50/HM** | 0.118 | -0.44 | 0.127 | -0.385 | -0.451 | -0.409 |
| **p21816-H50/HM** | 0.443 | -0.435 | 0.446 | -0.433 | -0.494 | -0.498 |
| **p21723-H50/HM** | 0.422 | -0.421 | 0.42 | -0.415 | -0.476 | -0.476 |
| **p29027-H50/LM** | -0.334 | 0.594 | -0.369 | 0.504 | 0.584 | 0.596 |
| **p28303-H50/LM** | -0.351 | 0.506 | -0.381 | 0.515 | 0.575 | 0.499 |
| **p28134-H50/LM** | -0.348 | 0.501 | -0.372 | 0.52 | 0.573 | 0.504 |
| **p14515-H50/LM** | 0.016 | 0.481 | 0.002 | 0.326 | 0.396 | 0.467 |
| **p14166-H50/LM** | -0.34 | 0.493 | -0.37 | 0.505 | 0.557 | 0.48 |
| **p14060-H50/LM** | -0.341 | 0.491 | -0.362 | 0.509 | 0.554 | 0.486 |
| **p7681-H50/LM** | 0.633 | -0.446 | 0.653 | -0.41 | -0.492 | -0.458 |
| **p6825-H50/LM** | 0.209 | -0.508 | 0.268 | -0.426 | -0.502 | -0.469 |
| **p6616-H50/LM** | 0.197 | -0.502 | 0.261 | -0.407 | -0.483 | -0.453 |
| **p6416-H50/LM** | 0.156 | -0.449 | 0.21 | -0.326 | -0.389 | -0.37 |
| **p3267-H50/LM** | 0.194 | -0.486 | 0.238 | -0.394 | -0.476 | -0.45 |
| **p89245-CM10/HM** | -0.014 | -0.571 | 0.039 | -0.632 | -0.645 | -0.637 |
| **p56259-CM10/HM** | -0.326 | 0.785 | -0.407 | 0.933 | 0.932 | 0.85 |
| **p29188-CM10/HM** | -0.353 | 0.8 | -0.43 | 0.917 | 0.942 | 0.984 |
| **p29000-CM10/HM** | -0.32 | 0.786 | -0.398 | 0.867 | 0.877 | 1 |
| **p28505-CM10/HM** | -0.384 | 0.743 | -0.456 | 0.973 | 1 | 0.877 |
| **p28321-CM10/HM** | -0.337 | 0.694 | -0.407 | 1 | 0.973 | 0.867 |
| **p20508-CM10/HM** | 0.95 | -0.365 | 1 | -0.407 | -0.456 | -0.398 |
| **p29024-CM10/LM** | -0.262 | 1 | -0.365 | 0.694 | 0.743 | 0.786 |
| **p20525-CM10/LM** | 1 | -0.262 | 0.95 | -0.337 | -0.384 | -0.32 |
| **p15150-CM10/LM** | -0.135 | 0.212 | -0.158 | 0.134 | 0.156 | 0.119 |
| **p14511-CM10/LM** | -0.294 | 0.946 | -0.395 | 0.62 | 0.677 | 0.786 |
| **p14151-CM10/LM** | -0.4 | 0.753 | -0.471 | 0.51 | 0.572 | 0.524 |
| **p14064-CM10/LM** | -0.239 | 0.942 | -0.34 | 0.727 | 0.735 | 0.705 |
| **p12770-CM10/LM** | 0.047 | -0.396 | 0.078 | -0.373 | -0.397 | -0.376 |
| **p8645-CM10/LM** | 0.192 | -0.432 | 0.23 | -0.389 | -0.41 | -0.405 |

|  | **p29188-CM10/HM** | **p56259-CM10/HM** | **p89245-CM10/HM** | **p3267-H50/LM** | **p6416-H50/LM** | **p6616-H50/LM** |
| --- | --- | --- | --- | --- | --- | --- |
| **p142405-H50/HM** | 0.556 | 0.481 | -0.341 | -0.597 | -0.633 | -0.712 |
| **p113729-H50/HM** | 0.582 | 0.515 | -0.337 | -0.613 | -0.637 | -0.726 |
| **p84946-H50/HM** | 0.565 | 0.51 | -0.298 | -0.585 | -0.602 | -0.692 |
| **p71729-H50/HM** | -0.47 | -0.383 | 0.379 | 0.437 | 0.278 | 0.357 |
| **p57201-H50/HM** | 0.65 | 0.502 | -0.318 | -0.618 | -0.579 | -0.681 |
| **p56387-H50/HM** | 0.535 | 0.492 | -0.226 | -0.496 | -0.462 | -0.548 |
| **p43483-H50/HM** | -0.498 | -0.414 | 0.328 | 0.421 | 0.244 | 0.328 |
| **p35840-H50/HM** | -0.566 | -0.488 | 0.323 | 0.575 | 0.543 | 0.655 |
| **p29188-H50/HM** | 0.578 | 0.371 | -0.163 | -0.346 | -0.305 | -0.368 |
| **p28992-H50/HM** | 0.572 | 0.311 | -0.163 | -0.317 | -0.241 | -0.311 |
| **p28523-H50/HM** | 0.386 | 0.352 | -0.082 | -0.327 | -0.383 | -0.443 |
| **p24017-H50/HM** | -0.448 | -0.362 | 0.2 | 0.432 | 0.446 | 0.486 |
| **p23922-H50/HM** | -0.455 | -0.371 | 0.261 | 0.486 | 0.559 | 0.589 |
| **p22752-H50/HM** | -0.452 | -0.438 | 0.484 | 0.623 | 0.499 | 0.579 |
| **p21816-H50/HM** | -0.528 | -0.434 | 0.28 | 0.418 | 0.242 | 0.321 |
| **p21723-H50/HM** | -0.508 | -0.421 | 0.282 | 0.413 | 0.227 | 0.308 |
| **p29027-H50/LM** | 0.63 | 0.525 | -0.423 | -0.764 | -0.65 | -0.761 |
| **p28303-H50/LM** | 0.554 | 0.529 | -0.402 | -0.754 | -0.64 | -0.748 |
| **p28134-H50/LM** | 0.556 | 0.535 | -0.4 | -0.745 | -0.589 | -0.7 |
| **p14515-H50/LM** | 0.479 | 0.385 | -0.471 | -0.696 | -0.554 | -0.671 |
| **p14166-H50/LM** | 0.533 | 0.516 | -0.382 | -0.717 | -0.617 | -0.722 |
| **p14060-H50/LM** | 0.536 | 0.521 | -0.38 | -0.7 | -0.542 | -0.648 |
| **p7681-H50/LM** | -0.504 | -0.416 | 0.199 | 0.405 | 0.488 | 0.534 |
| **p6825-H50/LM** | -0.507 | -0.478 | 0.445 | 0.884 | 0.857 | 0.978 |
| **p6616-H50/LM** | -0.49 | -0.461 | 0.426 | 0.851 | 0.887 | 1 |
| **p6416-H50/LM** | -0.4 | -0.368 | 0.342 | 0.698 | 1 | 0.887 |
| **p3267-H50/LM** | -0.492 | -0.451 | 0.478 | 1 | 0.698 | 0.851 |
| **p89245-CM10/HM** | -0.663 | -0.651 | 1 | 0.478 | 0.342 | 0.426 |
| **p56259-CM10/HM** | 0.895 | 1 | -0.651 | -0.451 | -0.368 | -0.461 |
| **p29188-CM10/HM** | 1 | 0.895 | -0.663 | -0.492 | -0.4 | -0.49 |
| **p29000-CM10/HM** | 0.984 | 0.85 | -0.637 | -0.45 | -0.37 | -0.453 |
| **p28505-CM10/HM** | 0.942 | 0.932 | -0.645 | -0.476 | -0.389 | -0.483 |
| **p28321-CM10/HM** | 0.917 | 0.933 | -0.632 | -0.394 | -0.326 | -0.407 |
| **p20508-CM10/HM** | -0.43 | -0.407 | 0.039 | 0.238 | 0.21 | 0.261 |
| **p29024-CM10/LM** | 0.8 | 0.785 | -0.571 | -0.486 | -0.449 | -0.502 |
| **p20525-CM10/LM** | -0.353 | -0.326 | -0.014 | 0.194 | 0.156 | 0.197 |
| **p15150-CM10/LM** | 0.14 | 0.133 | -0.101 | -0.113 | -0.058 | -0.064 |
| **p14511-CM10/LM** | 0.777 | 0.711 | -0.507 | -0.47 | -0.432 | -0.489 |
| **p14151-CM10/LM** | 0.558 | 0.597 | -0.343 | -0.397 | -0.387 | -0.425 |
| **p14064-CM10/LM** | 0.733 | 0.79 | -0.526 | -0.387 | -0.392 | -0.421 |
| **p12770-CM10/LM** | -0.387 | -0.421 | 0.444 | 0.379 | 0.225 | 0.345 |
| **p8645-CM10/LM** | -0.414 | -0.44 | 0.391 | 0.335 | 0.196 | 0.307 |

|  | **p6825-H50/LM** | **p7681-H50/LM** | **p14060-H50/LM** | **p14166-H50/LM** | **p14515-H50/LM** | **p28134-H50/LM** |
| --- | --- | --- | --- | --- | --- | --- |
| **p142405-H50/HM** | -0.688 | -0.667 | 0.685 | 0.738 | 0.6 | 0.728 |
| **p113729-H50/HM** | -0.705 | -0.695 | 0.713 | 0.759 | 0.586 | 0.757 |
| **p84946-H50/HM** | -0.683 | -0.679 | 0.722 | 0.756 | 0.505 | 0.762 |
| **p71729-H50/HM** | 0.407 | 0.4 | -0.302 | -0.281 | -0.32 | -0.336 |
| **p57201-H50/HM** | -0.685 | -0.743 | 0.666 | 0.704 | 0.559 | 0.702 |
| **p56387-H50/HM** | -0.565 | -0.628 | 0.672 | 0.67 | 0.344 | 0.698 |
| **p43483-H50/HM** | 0.387 | 0.433 | -0.332 | -0.305 | -0.224 | -0.363 |
| **p35840-H50/HM** | 0.648 | 0.619 | -0.585 | -0.632 | -0.644 | -0.619 |
| **p29188-H50/HM** | -0.381 | -0.638 | 0.403 | 0.427 | 0.297 | 0.412 |
| **p28992-H50/HM** | -0.327 | -0.496 | 0.365 | 0.359 | 0.357 | 0.359 |
| **p28523-H50/HM** | -0.41 | -0.592 | 0.529 | 0.597 | 0.192 | 0.534 |
| **p24017-H50/HM** | 0.516 | 0.42 | -0.467 | -0.495 | -0.171 | -0.494 |
| **p23922-H50/HM** | 0.593 | 0.443 | -0.514 | -0.563 | -0.304 | -0.545 |
| **p22752-H50/HM** | 0.595 | 0.431 | -0.467 | -0.482 | -0.5 | -0.498 |
| **p21816-H50/HM** | 0.388 | 0.488 | -0.394 | -0.357 | -0.189 | -0.423 |
| **p21723-H50/HM** | 0.368 | 0.473 | -0.357 | -0.326 | -0.183 | -0.388 |
| **p29027-H50/LM** | -0.762 | -0.632 | 0.898 | 0.93 | 0.822 | 0.925 |
| **p28303-H50/LM** | -0.747 | -0.588 | 0.959 | 0.984 | 0.749 | 0.987 |
| **p28134-H50/LM** | -0.715 | -0.559 | 0.977 | 0.97 | 0.733 | 1 |
| **p14515-H50/LM** | -0.66 | -0.349 | 0.737 | 0.764 | 1 | 0.733 |
| **p14166-H50/LM** | -0.72 | -0.558 | 0.975 | 1 | 0.764 | 0.97 |
| **p14060-H50/LM** | -0.67 | -0.51 | 1 | 0.975 | 0.737 | 0.977 |
| **p7681-H50/LM** | 0.52 | 1 | -0.51 | -0.558 | -0.349 | -0.559 |
| **p6825-H50/LM** | 1 | 0.52 | -0.67 | -0.72 | -0.66 | -0.715 |
| **p6616-H50/LM** | 0.978 | 0.534 | -0.648 | -0.722 | -0.671 | -0.7 |
| **p6416-H50/LM** | 0.857 | 0.488 | -0.542 | -0.617 | -0.554 | -0.589 |
| **p3267-H50/LM** | 0.884 | 0.405 | -0.7 | -0.717 | -0.696 | -0.745 |
| **p89245-CM10/HM** | 0.445 | 0.199 | -0.38 | -0.382 | -0.471 | -0.4 |
| **p56259-CM10/HM** | -0.478 | -0.416 | 0.521 | 0.516 | 0.385 | 0.535 |
| **p29188-CM10/HM** | -0.507 | -0.504 | 0.536 | 0.533 | 0.479 | 0.556 |
| **p29000-CM10/HM** | -0.469 | -0.458 | 0.486 | 0.48 | 0.467 | 0.504 |
| **p28505-CM10/HM** | -0.502 | -0.492 | 0.554 | 0.557 | 0.396 | 0.573 |
| **p28321-CM10/HM** | -0.426 | -0.41 | 0.509 | 0.505 | 0.326 | 0.52 |
| **p20508-CM10/HM** | 0.268 | 0.653 | -0.362 | -0.37 | 0.002 | -0.372 |
| **p29024-CM10/LM** | -0.508 | -0.446 | 0.491 | 0.493 | 0.481 | 0.501 |
| **p20525-CM10/LM** | 0.209 | 0.633 | -0.341 | -0.34 | 0.016 | -0.348 |
| **p15150-CM10/LM** | -0.076 | -0.124 | 0.097 | 0.09 | 0.114 | 0.089 |
| **p14511-CM10/LM** | -0.49 | -0.464 | 0.454 | 0.466 | 0.501 | 0.47 |
| **p14151-CM10/LM** | -0.429 | -0.446 | 0.404 | 0.421 | 0.283 | 0.424 |
| **p14064-CM10/LM** | -0.42 | -0.367 | 0.459 | 0.461 | 0.375 | 0.457 |
| **p12770-CM10/LM** | 0.367 | 0.102 | -0.217 | -0.207 | -0.29 | -0.236 |
| **p8645-CM10/LM** | 0.328 | 0.211 | -0.181 | -0.173 | -0.226 | -0.201 |

|  | **p28303-H50/LM** | **p29027-H50/LM** | **p21723-H50/HM** | **p21816-H50/HM** | **p22752-H50/HM** | **p23922-H50/HM** |
| --- | --- | --- | --- | --- | --- | --- |
| **p142405-H50/HM** | 0.76 | 0.79 | -0.426 | -0.449 | -0.532 | -0.631 |
| **p113729-H50/HM** | 0.783 | 0.795 | -0.468 | -0.49 | -0.538 | -0.653 |
| **p84946-H50/HM** | 0.778 | 0.754 | -0.511 | -0.538 | -0.483 | -0.673 |
| **p71729-H50/HM** | -0.317 | -0.408 | 0.88 | 0.859 | 0.47 | 0.215 |
| **p57201-H50/HM** | 0.727 | 0.823 | -0.633 | -0.66 | -0.565 | -0.714 |
| **p56387-H50/HM** | 0.688 | 0.637 | -0.61 | -0.639 | -0.4 | -0.63 |
| **p43483-H50/HM** | -0.34 | -0.409 | 0.98 | 0.965 | 0.433 | 0.284 |
| **p35840-H50/HM** | -0.643 | -0.696 | 0.357 | 0.377 | 0.442 | 0.454 |
| **p29188-H50/HM** | 0.437 | 0.596 | -0.625 | -0.648 | -0.42 | -0.603 |
| **p28992-H50/HM** | 0.362 | 0.559 | -0.562 | -0.59 | -0.359 | -0.51 |
| **p28523-H50/HM** | 0.59 | 0.539 | -0.387 | -0.401 | -0.339 | -0.639 |
| **p24017-H50/HM** | -0.52 | -0.545 | 0.381 | 0.435 | 0.461 | 0.936 |
| **p23922-H50/HM** | -0.585 | -0.607 | 0.334 | 0.376 | 0.551 | 1 |
| **p22752-H50/HM** | -0.516 | -0.574 | 0.432 | 0.43 | 1 | 0.551 |
| **p21816-H50/HM** | -0.396 | -0.461 | 0.99 | 1 | 0.43 | 0.376 |
| **p21723-H50/HM** | -0.365 | -0.429 | 1 | 0.99 | 0.432 | 0.334 |
| **p29027-H50/LM** | 0.945 | 1 | -0.429 | -0.461 | -0.574 | -0.607 |
| **p28303-H50/LM** | 1 | 0.945 | -0.365 | -0.396 | -0.516 | -0.585 |
| **p28134-H50/LM** | 0.987 | 0.925 | -0.388 | -0.423 | -0.498 | -0.545 |
| **p14515-H50/LM** | 0.749 | 0.822 | -0.183 | -0.189 | -0.5 | -0.304 |
| **p14166-H50/LM** | 0.984 | 0.93 | -0.326 | -0.357 | -0.482 | -0.563 |
| **p14060-H50/LM** | 0.959 | 0.898 | -0.357 | -0.394 | -0.467 | -0.514 |
| **p7681-H50/LM** | -0.588 | -0.632 | 0.473 | 0.488 | 0.431 | 0.443 |
| **p6825-H50/LM** | -0.747 | -0.762 | 0.368 | 0.388 | 0.595 | 0.593 |
| **p6616-H50/LM** | -0.748 | -0.761 | 0.308 | 0.321 | 0.579 | 0.589 |
| **p6416-H50/LM** | -0.64 | -0.65 | 0.227 | 0.242 | 0.499 | 0.559 |
| **p3267-H50/LM** | -0.754 | -0.764 | 0.413 | 0.418 | 0.623 | 0.486 |
| **p89245-CM10/HM** | -0.402 | -0.423 | 0.282 | 0.28 | 0.484 | 0.261 |
| **p56259-CM10/HM** | 0.529 | 0.525 | -0.421 | -0.434 | -0.438 | -0.371 |
| **p29188-CM10/HM** | 0.554 | 0.63 | -0.508 | -0.528 | -0.452 | -0.455 |
| **p29000-CM10/HM** | 0.499 | 0.596 | -0.476 | -0.498 | -0.409 | -0.431 |
| **p28505-CM10/HM** | 0.575 | 0.584 | -0.476 | -0.494 | -0.451 | -0.451 |
| **p28321-CM10/HM** | 0.515 | 0.504 | -0.415 | -0.433 | -0.385 | -0.401 |
| **p20508-CM10/HM** | -0.381 | -0.369 | 0.42 | 0.446 | 0.127 | 0.338 |
| **p29024-CM10/LM** | 0.506 | 0.594 | -0.421 | -0.435 | -0.44 | -0.398 |
| **p20525-CM10/LM** | -0.351 | -0.334 | 0.422 | 0.443 | 0.118 | 0.31 |
| **p15150-CM10/LM** | 0.096 | 0.139 | -0.114 | -0.105 | -0.144 | -0.072 |
| **p14511-CM10/LM** | 0.48 | 0.594 | -0.4 | -0.42 | -0.389 | -0.388 |
| **p14151-CM10/LM** | 0.434 | 0.462 | -0.328 | -0.348 | -0.271 | -0.337 |
| **p14064-CM10/LM** | 0.463 | 0.5 | -0.334 | -0.349 | -0.364 | -0.363 |
| **p12770-CM10/LM** | -0.229 | -0.229 | 0.17 | 0.16 | 0.328 | 0.167 |
| **p8645-CM10/LM** | -0.196 | -0.23 | 0.199 | 0.195 | 0.291 | 0.149 |

|  | **p24017-H50/HM** | **p28523-H50/HM** | **p28992-H50/HM** | **p29188-H50/HM** | **p35840-H50/HM** | **p43483-H50/HM** |
| --- | --- | --- | --- | --- | --- | --- |
| **p142405-H50/HM** | -0.555 | 0.576 | 0.475 | 0.545 | -0.688 | -0.418 |
| **p113729-H50/HM** | -0.577 | 0.608 | 0.484 | 0.552 | -0.729 | -0.463 |
| **p84946-H50/HM** | -0.617 | 0.652 | 0.468 | 0.544 | -0.698 | -0.496 |
| **p71729-H50/HM** | 0.197 | -0.222 | -0.455 | -0.498 | 0.438 | 0.934 |
| **p57201-H50/HM** | -0.664 | 0.684 | 0.757 | 0.829 | -0.722 | -0.61 |
| **p56387-H50/HM** | -0.613 | 0.65 | 0.478 | 0.551 | -0.593 | -0.578 |
| **p43483-H50/HM** | 0.31 | -0.328 | -0.519 | -0.569 | 0.385 | 1 |
| **p35840-H50/HM** | 0.335 | -0.447 | -0.391 | -0.465 | 1 | 0.385 |
| **p29188-H50/HM** | -0.616 | 0.675 | 0.924 | 1 | -0.465 | -0.569 |
| **p28992-H50/HM** | -0.535 | 0.452 | 1 | 0.924 | -0.391 | -0.519 |
| **p28523-H50/HM** | -0.593 | 1 | 0.452 | 0.675 | -0.447 | -0.328 |
| **p24017-H50/HM** | 1 | -0.593 | -0.535 | -0.616 | 0.335 | 0.31 |
| **p23922-H50/HM** | 0.936 | -0.639 | -0.51 | -0.603 | 0.454 | 0.284 |
| **p22752-H50/HM** | 0.461 | -0.339 | -0.359 | -0.42 | 0.442 | 0.433 |
| **p21816-H50/HM** | 0.435 | -0.401 | -0.59 | -0.648 | 0.377 | 0.965 |
| **p21723-H50/HM** | 0.381 | -0.387 | -0.562 | -0.625 | 0.357 | 0.98 |
| **p29027-H50/LM** | -0.545 | 0.539 | 0.559 | 0.596 | -0.696 | -0.409 |
| **p28303-H50/LM** | -0.52 | 0.59 | 0.362 | 0.437 | -0.643 | -0.34 |
| **p28134-H50/LM** | -0.494 | 0.534 | 0.359 | 0.412 | -0.619 | -0.363 |
| **p14515-H50/LM** | -0.171 | 0.192 | 0.357 | 0.297 | -0.644 | -0.224 |
| **p14166-H50/LM** | -0.495 | 0.597 | 0.359 | 0.427 | -0.632 | -0.305 |
| **p14060-H50/LM** | -0.467 | 0.529 | 0.365 | 0.403 | -0.585 | -0.332 |
| **p7681-H50/LM** | 0.42 | -0.592 | -0.496 | -0.638 | 0.619 | 0.433 |
| **p6825-H50/LM** | 0.516 | -0.41 | -0.327 | -0.381 | 0.648 | 0.387 |
| **p6616-H50/LM** | 0.486 | -0.443 | -0.311 | -0.368 | 0.655 | 0.328 |
| **p6416-H50/LM** | 0.446 | -0.383 | -0.241 | -0.305 | 0.543 | 0.244 |
| **p3267-H50/LM** | 0.432 | -0.327 | -0.317 | -0.346 | 0.575 | 0.421 |
| **p89245-CM10/HM** | 0.2 | -0.082 | -0.163 | -0.163 | 0.323 | 0.328 |
| **p56259-CM10/HM** | -0.362 | 0.352 | 0.311 | 0.371 | -0.488 | -0.414 |
| **p29188-CM10/HM** | -0.448 | 0.386 | 0.572 | 0.578 | -0.566 | -0.498 |
| **p29000-CM10/HM** | -0.434 | 0.329 | 0.621 | 0.582 | -0.519 | -0.466 |
| **p28505-CM10/HM** | -0.439 | 0.454 | 0.391 | 0.477 | -0.538 | -0.462 |
| **p28321-CM10/HM** | -0.394 | 0.418 | 0.334 | 0.409 | -0.464 | -0.399 |
| **p20508-CM10/HM** | 0.435 | -0.616 | -0.463 | -0.595 | 0.236 | 0.345 |
| **p29024-CM10/LM** | -0.371 | 0.299 | 0.486 | 0.482 | -0.474 | -0.418 |
| **p20525-CM10/LM** | 0.397 | -0.616 | -0.461 | -0.593 | 0.194 | 0.342 |
| **p15150-CM10/LM** | -0.068 | 0.071 | 0.188 | 0.208 | -0.051 | -0.094 |
| **p14511-CM10/LM** | -0.373 | 0.277 | 0.543 | 0.514 | -0.497 | -0.393 |
| **p14151-CM10/LM** | -0.351 | 0.305 | 0.327 | 0.375 | -0.347 | -0.306 |
| **p14064-CM10/LM** | -0.335 | 0.299 | 0.341 | 0.37 | -0.386 | -0.325 |
| **p12770-CM10/LM** | 0.145 | 0.058 | -0.068 | -0.033 | 0.116 | 0.196 |
| **p8645-CM10/LM** | 0.17 | 0.021 | -0.169 | -0.144 | 0.094 | 0.209 |

|  | **p56387-H50/HM** | **p57201-H50/HM** | **p71729-H50/HM** | **p84946-H50/HM** | **p113729-H50/HM** | **p142405-H50/HM** |
| --- | --- | --- | --- | --- | --- | --- |
| **p142405-H50/HM** | 0.788 | 0.847 | -0.367 | 0.919 | 0.964 | 1 |
| **p113729-H50/HM** | 0.868 | 0.885 | -0.403 | 0.972 | 1 | 0.964 |
| **p84946-H50/HM** | 0.948 | 0.876 | -0.395 | 1 | 0.972 | 0.919 |
| **p71729-H50/HM** | -0.429 | -0.549 | 1 | -0.395 | -0.403 | -0.367 |
| **p57201-H50/HM** | 0.823 | 1 | -0.549 | 0.876 | 0.885 | 0.847 |
| **p56387-H50/HM** | 1 | 0.823 | -0.429 | 0.948 | 0.868 | 0.788 |
| **p43483-H50/HM** | -0.578 | -0.61 | 0.934 | -0.496 | -0.463 | -0.418 |
| **p35840-H50/HM** | -0.593 | -0.722 | 0.438 | -0.698 | -0.729 | -0.688 |
| **p29188-H50/HM** | 0.551 | 0.829 | -0.498 | 0.544 | 0.552 | 0.545 |
| **p28992-H50/HM** | 0.478 | 0.757 | -0.455 | 0.468 | 0.484 | 0.475 |
| **p28523-H50/HM** | 0.65 | 0.684 | -0.222 | 0.652 | 0.608 | 0.576 |
| **p24017-H50/HM** | -0.613 | -0.664 | 0.197 | -0.617 | -0.577 | -0.555 |
| **p23922-H50/HM** | -0.63 | -0.714 | 0.215 | -0.673 | -0.653 | -0.631 |
| **p22752-H50/HM** | -0.4 | -0.565 | 0.47 | -0.483 | -0.538 | -0.532 |
| **p21816-H50/HM** | -0.639 | -0.66 | 0.859 | -0.538 | -0.49 | -0.449 |
| **p21723-H50/HM** | -0.61 | -0.633 | 0.88 | -0.511 | -0.468 | -0.426 |
| **p29027-H50/LM** | 0.637 | 0.823 | -0.408 | 0.754 | 0.795 | 0.79 |
| **p28303-H50/LM** | 0.688 | 0.727 | -0.317 | 0.778 | 0.783 | 0.76 |
| **p28134-H50/LM** | 0.698 | 0.702 | -0.336 | 0.762 | 0.757 | 0.728 |
| **p14515-H50/LM** | 0.344 | 0.559 | -0.32 | 0.505 | 0.586 | 0.6 |
| **p14166-H50/LM** | 0.67 | 0.704 | -0.281 | 0.756 | 0.759 | 0.738 |
| **p14060-H50/LM** | 0.672 | 0.666 | -0.302 | 0.722 | 0.713 | 0.685 |
| **p7681-H50/LM** | -0.628 | -0.743 | 0.4 | -0.679 | -0.695 | -0.667 |
| **p6825-H50/LM** | -0.565 | -0.685 | 0.407 | -0.683 | -0.705 | -0.688 |
| **p6616-H50/LM** | -0.548 | -0.681 | 0.357 | -0.692 | -0.726 | -0.712 |
| **p6416-H50/LM** | -0.462 | -0.579 | 0.278 | -0.602 | -0.637 | -0.633 |
| **p3267-H50/LM** | -0.496 | -0.618 | 0.437 | -0.585 | -0.613 | -0.597 |
| **p89245-CM10/HM** | -0.226 | -0.318 | 0.379 | -0.298 | -0.337 | -0.341 |
| **p56259-CM10/HM** | 0.492 | 0.502 | -0.383 | 0.51 | 0.515 | 0.481 |
| **p29188-CM10/HM** | 0.535 | 0.65 | -0.47 | 0.565 | 0.582 | 0.556 |
| **p29000-CM10/HM** | 0.489 | 0.618 | -0.438 | 0.518 | 0.537 | 0.516 |
| **p28505-CM10/HM** | 0.554 | 0.592 | -0.418 | 0.573 | 0.572 | 0.536 |
| **p28321-CM10/HM** | 0.51 | 0.502 | -0.352 | 0.517 | 0.506 | 0.469 |
| **p20508-CM10/HM** | -0.579 | -0.556 | 0.203 | -0.503 | -0.447 | -0.4 |
| **p29024-CM10/LM** | 0.408 | 0.59 | -0.416 | 0.469 | 0.514 | 0.508 |
| **p20525-CM10/LM** | -0.579 | -0.538 | 0.2 | -0.492 | -0.431 | -0.386 |
| **p15150-CM10/LM** | 0.041 | 0.181 | -0.113 | 0.059 | 0.082 | 0.096 |
| **p14511-CM10/LM** | 0.422 | 0.612 | -0.38 | 0.482 | 0.524 | 0.52 |
| **p14151-CM10/LM** | 0.388 | 0.479 | -0.267 | 0.424 | 0.427 | 0.413 |
| **p14064-CM10/LM** | 0.377 | 0.48 | -0.31 | 0.423 | 0.453 | 0.44 |
| **p12770-CM10/LM** | -0.097 | -0.13 | 0.233 | -0.125 | -0.138 | -0.132 |
| **p8645-CM10/LM** | -0.115 | -0.177 | 0.216 | -0.141 | -0.167 | -0.162 |

Each ion peak detected is named using the initial “p” followed by its m/z value, the type of array on which it was detected (H50 or CM10) and then the laser intensity: low mass (LM) or high mass (HM).
