# Supplementary material for: Multimarker Proteomic Profiling for the Prediction of Cardiovascular Mortality in Patients with Chronic Heart Failure
Source: PLoS One. 2015 Apr 23;10(4):e0119265. doi: 10.1371/journal.pone.0119265 (PMC4408082; doi:10.1371/journal.pone.0119265)
Supplement: S3 Table — The proteomic scores were developed using the support vector machine (SVM), the sparse partial least square discriminant analysis (sPLS-DA) and the lasso logistic regression (LASSO). (DOC) [file pone.0119265.s005.doc]

###### Table S3. Pearson correlation matrix of the proteomic scores in the discovery population

| Methods | SVM | sPLS-DA | LASSO |
| --- | --- | --- | --- |
| SVM | 1 | 0.88 | 0.94 |
| sPLS-DA | 0.88 | 1 | 0.94 |
| LASSO | 0.94 | 0.94 | 1 |

The proteomic scores were developed using the support vector machine (SVM), the sparse partial least square discriminant analysis (sPLS-DA) and the lasso logistic regression (LASSO).
