# Supplementary figures and images for: Multimarker Proteomic Profiling for the Prediction of Cardiovascular Mortality in Patients with Chronic Heart Failure
Source: PLoS One. 2015 Apr 23;10(4):e0119265. doi: 10.1371/journal.pone.0119265 (PMC4408082; doi:10.1371/journal.pone.0119265)

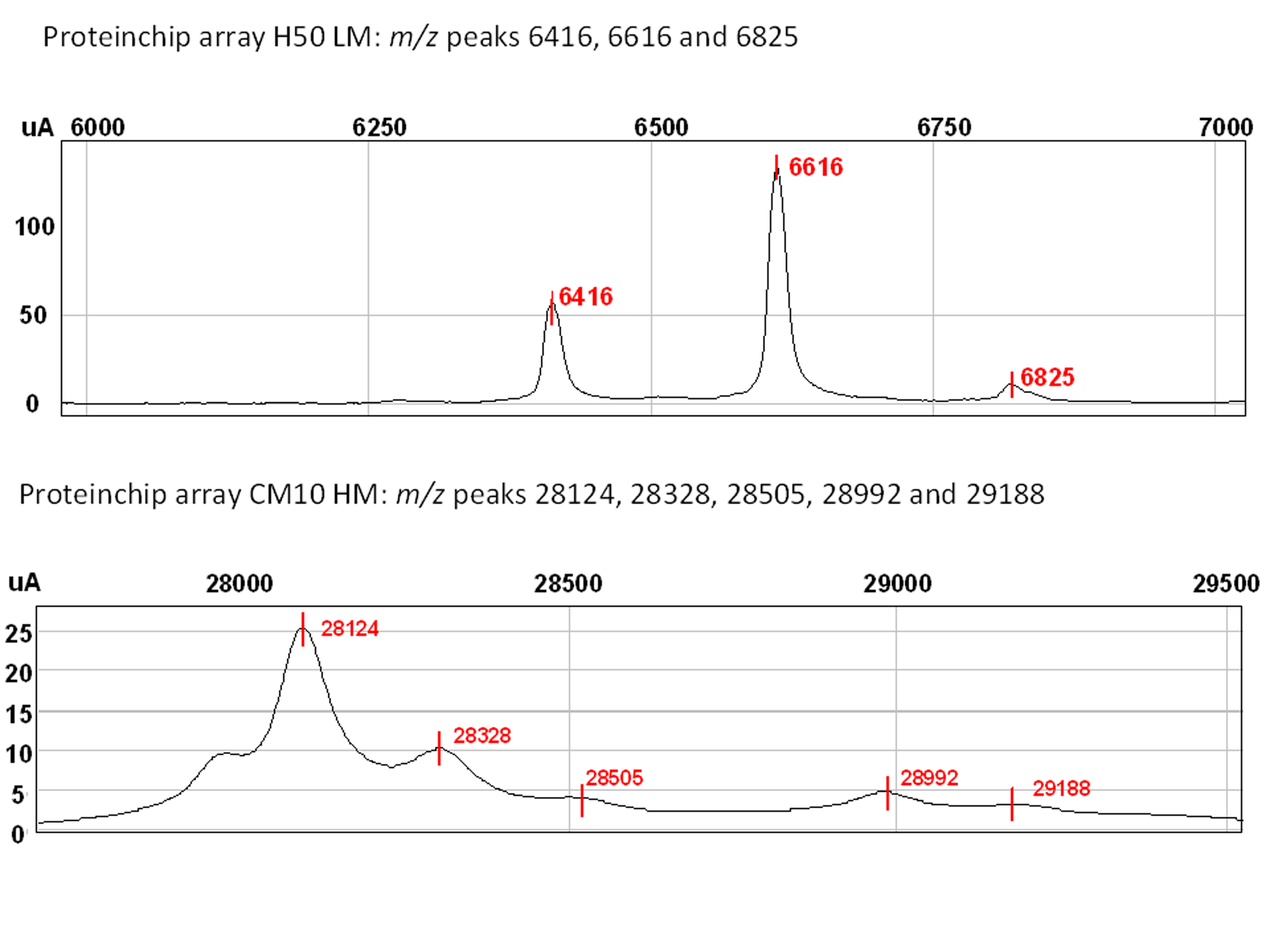

Supplement: S1 Fig — The upper mass spectrum was obtained from the H50 proteinchip array using low-mass (LM) parameter settings and the lower mass spectrum was obtained from the CM10 proteinchip array using high-mass (HM) parameter settings. (TIF) [file pone.0119265.s006.TIF]
